# Comparison of the Nodule *vs.* Root Transcriptome of the Actinorhizal Plant *Datisca glomerata:* Actinorhizal Nodules Contain a Specific Class of Defensins

**DOI:** 10.1371/journal.pone.0072442

**Published:** 2013-08-29

**Authors:** Irina V. Demina, Tomas Persson, Patricia Santos, Marian Plaszczyca, Katharina Pawlowski

**Affiliations:** 1 Department of Botany, Stockholm University, Stockholm, Sweden; 2 Department of Plant Pathology, Michigan State University, East Lansing, Michigan, United States of America; Justus-Liebig-University Giessen, Germany

## Abstract

Actinorhizal root nodule symbioses are very diverse, and the symbiosis of *Datisca glomerata* has previously been shown to have many unusual aspects. In order to gain molecular information on the infection mechanism, nodule development and nodule metabolism, we compared the transcriptomes of *D. glomerata* roots and nodules. Root and nodule libraries representing the 3′-ends of cDNAs were subjected to high-throughput parallel 454 sequencing. To identify the corresponding genes and to improve the assembly, Illumina sequencing of the nodule transcriptome was performed as well. The evaluation revealed 406 differentially regulated genes, 295 of which (72.7%) could be assigned a function based on homology. Analysis of the nodule transcriptome showed that genes encoding components of the common symbiosis signaling pathway were present in nodules of *D. glomerata,* which in combination with the previously established function of SymRK in *D. glomerata* nodulation suggests that this pathway is also active in actinorhizal Cucurbitales. Furthermore, comparison of the *D. glomerata* nodule transcriptome with nodule transcriptomes from actinorhizal Fagales revealed a new subgroup of nodule-specific defensins that might play a role specific to actinorhizal symbioses. The *D. glomerata* members of this defensin subgroup contain an acidic C-terminal domain that was never found in plant defensins before.

## Introduction

Two types of nitrogen-fixing root nodule symbioses are known: legume-rhizobia symbioses and actinorhizal symbioses. The actinorhizal symbiosis is a symbiosis between actinobacteria of the genus *Frankia* and a diverse group of dicotyledonous plant species from eight different families, collectively called actinorhizal plants [1]. Phylogenetic analysis led to the identification of three major subgroups of actinorhizal plants: Fagales (Betulaceae, Myricaceae and Casuarinaceae), Cucurbitales (Datiscaceae and Coriariaceae) and Rosales (Rhamnaceae, Rosaceae and Elaeagnaceae) [2].

Actinorhizal nodules are coralloid organs composed of multiple lobes, each of which represents a modified lateral root without root cap, with a superficial periderm and infected cells in the expanded cortex [3]. In nodules formed on the roots of Cucurbitales, the pattern of infected cells is different from that in other actinorhizal nodules; the infected cells form a continuous patch on one side of the acentric stele, not interspersed with uninfected cells [4], [5]. Nodules of Cucurbitales are unusual in other respects as well; nodule physiology [6], [7], anatomy [8], [9] and metabolism [10], [11], [12] of the best-examined member of actinorhizal Cucurbitales, *Datisca glomerata,* differ from those of actinorhizal nodules formed on Fagales or Rosales. The mechanism of nodule induction on roots of actinorhizal Cucurbitales has not been examined yet, but detailed cytological analyses of mature nodules of *D. glomerata* [12] have led to the conclusion that the mechanism by which the bacteria enter plant cells may be different from those found in actinorhizal Fagales (intracellular infection) and Rosales (intercellular infection), respectively [3]. The absence of prenodules in Cucurbitales would argue for the intercellular infection pathway, but the transcellular growth of infection threads for the intracellular pathway. Yet, in *D. glomerata* transcellular infection thread growth was not preceded by the formation of pre-infection thread structures [13]. In summary, actinorhizal Cucurbitales may have a unique mechanism for transcellular infection thread growth [3].

In order to understand nodule development, many studies have been conducted on the comparison of gene expression patterns in legume nodules *vs.* roots (e.g., [14], [15]). For actinorhizal plants, several differential screenings have been carried out (e.g., [16], [17], [18]); however, a large scale transcriptomics analysis has only been performed for *Casuarina glauca* and *Alnus glutinosa* (Fagales) [19], [20]. To date, most transcriptome studies have been conducted by microarray hybridization analysis, but the production of microarrays relies on information from extensive EST sequencing. With the reduced cost of sequencing, transcript profiling is becoming the standard technique for analysing both expression patterns [21] and quantitative traits [22]. Nevertheless, the short sequence reads of serial analysis of gene expression (SAGE) [23] and related techniques are severely limited by the requirement of a genome sequence with reliable annotation, which is not available for many plant species including *D. glomerata.* Use of the 454 GS FLX sequencing technology (Roche), which creates reads of 200 bp or more in length [24], [25], while providing a lower depth of sequencing compared to short-read technologies like Solexa 1-G, offers the possibility to yield sufficient sequence information to overcome this limitation. The parallel 454 sequencing method applies high-throughput sequencing for the use with multiple samples by attaching sample-specific barcoding adaptors to blunt-end repaired DNA samples by ligation and strand-displacement (Figure S1) [26]. With this procedure, 3′-anchored template cDNA libraries are constructed in order to generate gene-specific sequence reads. The 5′-end is generated by cutting with the restriction endonuclease *Nla*III.

The parallel 454 sequencing was used to obtain an overview of the *D. glomerata* root and nodule transcriptomes and to enable a comparison with other root nodule symbioses. However, even with this method only 13.4% of transcripts could be identified based on sequence homology. In order to improve the assembly and the identification of genes, Illumina sequencing of the nodule transcriptome was performed as well. With these data, transcript identification could be improved to reach 72.7%, leading to a better understanding of the similarities and differences between actinorhizal root nodules from Cucurbitales and Fagales, respectively.

## Results and Discussion

### Sequencing of SAGE-type libraries from roots and nodules of *Datisca glomerata*

In order to obtain an overview of the *D. glomerata* root and nodule transcriptome, a high-throughput method for sequencing of serial analysis of gene expression (SAGE)-type cDNA libraries by 454 GS FLX technology (Roche) was used [26]. 3′-End cDNA libraries were prepared from high quality total RNA from roots and nodules of *D. glomerata,* respectively (Figure S1). Altogether, 103,949 individual cDNA 3′-end sequences were obtained; of these 54,833 came from the root library and 49,116 from the nodule library. Clustering of these sequences led to 6,918 unique contigs (GenBank accession no. SRA012607.3).

The functions of the corresponding genes were analysed using BlastX searches of the contig sequences against the DNA databases at www.ncbi.nlm.nih.gov. Identification frequency was 15.4% when significant homology with database sequences was considered (e-value <10^−5^), 13.4% when homology with unknown/unidentified/hypothetical proteins was excluded. I.e., 930 contigs could be assigned homology to a gene/transcript from another organism or a gene previously characterized in *D. glomerata.*

The assembled length of the contigs varied: 983 contigs <100 bp, 1,900 contigs of 100–200 bp, 3,552 contigs of 200–300 bp and 483 contigs >400 bp. Statistical analysis was performed using the method described by Journet et al. [27] to decide whether the differences in the number of representatives in the root *vs.* the nodule SAGE-type library indicated significantly different expression levels. R = 10 was set as a threshold above which a difference observed was considered significant. 419 contigs were considered significantly upregulated and of these 86 had homology to transcripts in the databases at www.ncbi.nlm.nih.gov.

However, the number of 6,918 unique contigs was overrated because several contigs identified as unique in the assembly were shown to represent two previously characterized genes (see, e.g., *Dgc217* and *Dgc63,* Table S1A).

### Illumina sequencing of the nodule transcriptome and assembly

In order to improve the cDNA identification rate, the sequences had to be extended in the 5′-direction. 3′-UTR sequences tend to be AT-rich and generally are not suited to devise gene-specific primers for successful 5′-RACEs. Therefore, paired-end sequencing of the nodule transcriptome was performed using an Illumina HiSeq2000 instrument (NIH short read archive, study accession number SRP026310). Application of CLC Bio Workbench v. 4 (CLC Bio) resulted in the assembly of 117,511 contigs with an average length of 685 bp. Application of Trinity [28] resulted in the assembly of 64,142 contigs with an average length of 1,318 bp (N50 = 3,397 bp). This collection of sequences will be referred to as ”Trinity assembly“ in this manuscript. Then, CAP3 [29] was used to perform a ”meta-assembly”, in which the contigs of the Trinity assembly were combined with the contigs obtained from 454 sequencing. This resulted in a total of 9,180 contigs, 272 of which consisted only of assembled 454-contigs, while 3,128 were mixed contigs of Trinity and 454-origin and the remaining 5,780 contigs were based solely on Trinity contigs. This collection of sequences will be referred to as ”meta-assembly”.

The new sequence information was to be used to improve identification of the functions of the genes represented by 454-contigs and to determine which 454-contigs represented the same genes, and thereby to make the statistical analysis more reliable. The extended sequence length for 3,128 of the mixed contigs of the meta-assembly improved the identification of homologies *via* BlastX. A detailed analysis of 288 mixed contigs of the meta-assembly showed that 234 of them (81.35%) showed homology with database sequences (78.5% when homology with conserved proteins of unknown function was not included). Of these 288 meta-assembly contigs, only 15 (5.2%) were chimeric, i.e., they consisted of more than one cDNA based on homology analyses using blastX (data not shown). However, when 73 of the mixed meta-assembly contigs were analysed in detail, it was found that for 48 (55%) of them no significant sequence overlap existed with the 454-contigs that had supposedly been used for their assembly. In conclusion, the quality of the meta-assembly was too low to be used as a template to sort the 454-sequences, since the links between meta-assembly contigs and 454-contigs were not reliable.

In order to assess the possibility of using the Trinity assembly as a template for sorting the 454-sequences, blastN was used to compare all 6,918 of the 454-contigs to the Trinity contigs, and all results were quality-checked by eye. This resulted in 3,756 Trinity contigs or groups of Trinity contigs that represented 5,258 different 454-contigs and 6,936 different Trinity contigs, respectively. However, a blastX search for GenBank homologs of the 101 Trinity sequences with the highest R values revealed that 40 of them were chimeric, i.e., consisted of two or more cDNAs (five of the 101 Trinity contigs examined showed no homology to GenBank sequences and therefore could not be evaluated). In conclusion, the contribution of chimeric contigs in the Trinity assembly was far too high to use this assembly as a template for sorting the 454-sequences.

Therefore, the initial 454-assembly was improved for homology identification and for the identification of contigs representing the same gene, based on the added sequence information. The alignments of the 3,756 Trinity contigs with 454-contigs were checked individually and used as a basis to combine 454-contigs unambiguously derived from the same cDNA as 454-/Trinity supercontigs (Table S1A and S1B). For the 454-contigs with R>10, blastN searches were performed against the Trinity assembly, the meta-assembly and the 454-assembly to (a) get sequence extension for identification of gene functions and (b) improve the 454-assembly by finding more supercontigs. Poisson statistics was applied to this superassembly based on the occurrences of the original 454-contigs in the root and nodule SAGE-type libraries. This way, 406 genes were identified that were expressed differentially in nodules *vs.* roots of *D. glomerata.* 86.7% (352) of these genes showed homology to database sequences, which after subtraction of homologies with conserved proteins of unknown function resulted in an identification rate of 72.7% (Table S1A and S1B). 4.6% of the 454-contigs were not represented in either the Trinity assembly or the meta-assembly. All three assemblies are available for Blast searches at fido.nsc.liu.se.

Several *D. glomerata* genes the cDNAs of which had been characterized previously were found in the combined assemblies. The only result on transcription levels that contradicted previously published information was on *DgGHRP1* (*Dgc1* in Table S1B; glycine- and histidine-rich protein [30]). This gene had been published as showing nodule-enhanced expression based on RNA gel blot hybridization analysis and here appeared as root-enhanced. However, *in situ* hybridization had shown that *DgGHRP1* is expressed mainly in the periderm of roots and nodules, and the roots used for RNA isolation in this study were older than the roots used in the previous study (ca. 4 cm root length *vs.* 1 cm root length, starting at the tip). Hence, the difference in relative expression levels can be explained by the fact that the young root parts used in the previous study hardly contained any periderm, while the longer roots used in this study contained periderm and thus much more *DgGHRP1* mRNA.

### Confirmation of transcriptomics data by quantitative real-time PCR (qPCR) analysis

In order to analyse the reliability of the SAGE-type library sequencing method for determining relative levels of transcription, qPCR was performed for 23 genes for which homology with database sequences had been found. These analyses were performed on newly isolated RNAs from plants grown under the same conditions as those used for the isolation of RNA for the SAGE-type libraries, except in soil instead of sand. The results are depicted in Table 1. Altogether, with regard to tendency, applying t-test (p<0.05) to the qPCR results and setting a cut-off ratio of fold change as 2, confirmation of the SAGE-type library sequencing results was found for 73.9% of the genes examined, i.e., for 17 out of 23 genes. With regard to sensitivity/sequencing depth, organ-specificity (when set as fold change ≥100) could only be confirmed in six out of 14 cases. When taking the R values of the SAGE-type library results into consideration, it is clear that tendencies were always confirmed for R values >300, i.e., the results of SAGE-type library sequencing were basically reliable though with a higher cut-off value than would be expected based on modified Poisson statistics. The lack of confirmation of tendency for *DgMnSOD1* (*Dgc73*; Table S1A), a superoxide dismutase gene, might be explained by physiological differences of the root samples used for SAGE-type library construction *vs.* those used for qPCR. It can be expected that the expression of a superoxide dismutase is controlled by various abiotic stresses [31].

**Table 1:** Validation of SAGE-type library comparison results and analysis of expression levels of genes encoding proteins involved in infection thread formation by quantitative real-time polymerase chain reaction (qPCR).

Transcript	Encoded protein	Nr of cDNA reads Nodules Roots			R value	Fold change	Tendency*
Nodule-enhanced genes*							
***Dgc11***	Homolog of a soybean early nodulin gene, Gm93		91	0	9.30E+19	**3907.2**	confirmed
***Dgc156***	Defensin1 (DgDEF1)		88	0	2.00E+19	**2223.4**	confirmed
***Dgc232***	Cysteine-rich protein 1 (DgCRP1)		31	0	4.34E+06	**6834.6**	confirmed
***Dgc108***	Nitrate/oligopeptide/dicarboxylate transporter (DgDCAT1)		24	0	1.21E+05	**74027.1**	confirmed
***Dgc768***	Protein with unknown function		20	0	1.56E+04	**573.8**	confirmed
***Dgc26***	Snakin-2 (antimicrobial protein)		44	9	3081.5	**3.0**	confirmed
***Dgc148***	Glutamate decarboxylase		22	3	187.5	−1.2	not confirmed
***Dgc73***	DgMnSOD1		24	4	151.4	−1.2	not confirmed
***Dgc1305***	Syntaxin		10	0	93.7	1.8	not confirmed
***Dgc955***	Remorin2 (DgREM2)		8	0	22.8	**9.6**	confirmed
***Dgc845***	Defensin2 (DgDEF2)		7	0	20.2	**89.5**	confirmed
***Dgc1007***	Nodule inception protein (DgNIN1)		7	0	20.2	**6.8**	confirmed
***Dgc970***	Putative hybrid proline-rich protein		18	4	17.9	−2.4	not confirmed
***Dgc80***	Proline-rich glycoprotein, extensin, HyPRP1		18	4	17.9	−**4.5**	not confirmed
Root-enhanced genes*							
***Dgc39***	Cationic peroxidase		0	28	1.81E+06	−3.2	confirmed
**Dgc87**	Cysteine protease		1	16	280.8	−**1.9**	not confirmed
***Dgc670***	Xyloglucan endotransglycosylase/hydrolase XTH-3		0	10	118.6	−**2.0**	confirmed
***Dgc1083***	Indole-3-acetic acid amido synthetase		0	8	40.7	−**5.8**	confirmed
***Dgc1131***	Extensin-like protein (lipid binding)		0	7	23.8	−**195.2**	confirmed
***Dgc556***	Aquaporin PIP-1		1	10	17.3	−**4.4**	confirmed
***Dgc1139***	Ras-related small GTP-binding protein		0	6	14.0	−**4.0**	confirmed
***Dgc323***	Polygalacturonase		2	12	13.5	−**7.1**	confirmed
***Dgc54***	SNAP33; t-SNARE		9	25	13.1	−**3.0**	confirmed
***Dgc3012***	Vapyrin (DgVPY1)		0	4	4.8	**43.8**	not applicable†
Genes not induced in either roots or nodules*							
***Dgc390***	Remorin 1 (DgREM1)		8	8	0.8	1.2	not applicable†
***Dgc3716***	CERBERUS/LIN		2	2	1.6	−1.9	not applicable†
***comp11879***	PUB1 (DgPUB1)		-	-	-	−2.0	not applicable

Values for qPCR results represent means of three biological replicates.

* Contigs were originally classified based on 454 sequencing results. The tendency (nodule-enhanced or root-enhanced expression with R>10 based on the evaluation of the SAGE-type libraries) is considered to be confirmed when the fold change ≥2 (for nodule-enhanced genes) and ≤−2 (for root-enhanced genes) and the difference is significant, p<0.05. Bold indicates p<0.05; bold underline indicates p<0.0019 (Bonferroni correction).

† Due to R<10.

### Genes induced or downregulated in *D. glomerata* nodules compared to roots: overview

Analysis of the functions of the genes that are significantly up- or downregulated in nodules compared to roots implies that primary C and N metabolism is more complex in nodules than in roots, while secondary metabolism is more complex in roots (Table 2). There seem to be more transporter genes induced in nodules, presumably due to the fact that the induction of genes encoding transporters involved in nutrient exchange with the microsymbionts in nodules is higher than the induction of transporters involved in nutrient uptake from the soil in roots. The latter might easily be below the detection level. Interestingly, some chaperonins are induced in nodules, while none seems to be induced in roots, suggesting osmotic stress in nodules.

**Table 2:** Functions induced in nodules and roots, respectively; expression levels based on SAGE-type libraries.

	induced in nodules	induced in roots
**C metabolism**	23	9
**N metabolism**	9	0
**secondary metabolism**	4	16
**transcription factors**	9	13
**signal transduction**	16	17
**cell wall proteins**	3	7
**proteases**	4	6
**transporters**	8	5
**aquaporins**	1	4
**chaperons**	3	0
**cysteine-rich peptides**	5	1

### Homologs of genes encoding the components of the common symbiosis signal transduction pathway of legumes are transcribed in *D. glomerata* nodules

The signal transduction pathway of arbuscular mycorrhizal signal factors (Myc factors) [32] that was recruited for the evolution of root nodule symbiosis and therefore is also used by rhizobial nodulation (Nod) factors is referred to as the common symbiosis pathway. Results of Markmann et al. [33] have shown that a major component of this common symbiosis pathway of legumes, SymRK, is required for the induction of *D. glomerata* nodules by the homologous *Frankia* strain.

To find out whether genes encoding other components of the common symbiosis signal transduction pathway as well as other genes encoding proteins essential for legume nodule induction were present in the *D. glomerata* transcriptome, tblastN analyses were performed on the Trinity assembly and meta-assembly of the nodule transcriptome. The results showed that most legume genes encoding components of the common symbiosis pathway and genes essential for legume nodulation have homologs that are transcribed in *D. glomerata* nodules (Table 3). E.g., homologs of the LysM receptor kinases that act as Nod factor receptors [34] were found. Most of these signal transduction pathway component homologs (the nucleoporin NUP133, the calcium- and calmodulin-dependent protein kinase DMI3/CCaMK, the cation channels DMI1/CASTOR/POLLUX, the cytokinin receptor histidine kinase HK1, the transcription factor PIR1, IPD3/CYCLOPS and the transcription factor ERN1) were represented in the Trinity assembly, but not in the SAGE-type libraries (Table 3).

**Table 3:** *D. glomerata* homologs of genes encoding components of the common symbiosis pathway or proteins involved in infection thread growth in legumes (legume data from [34], [36], [39], [40], [42], [43], [44], [77], [78], [79], [80], [81], [82], [83], [84], [85], [86], [87], [88]).

Legume gene	Accession no. GenBank/EMBL	*D. glomerata* homolog in Trinity assembly	*D. glomerata* homologs in SAGE-type libraries	Reads in nodules roots		qPCR roots /nodules	Expression in legume roots/nodules
***NFR1/LYK3***	CAE02590	comp2293_c1_seq4	*Dgc4305*	2	0	n.d.	root-specific
***NFR5/NFP***	CAE02597	comp7396_c0_seq1	*Dgc5814*	2	0	n.d.	similar levels
***DMI2/SymRK***	CAP62376	comp941_c0_seq1	*Dgc4921*	0	2	n.d.	similar levels
***DMI3/CCaMK***	CAJ76700	comp5284_c0_seq1	-	-	-	n.d.	nodule-enhanced
***DMI1/CASTOR*** ***/POLLUX***	BAD89021/ BAD89022	comp5284_c0_seq1, comp9681_c0_seq10, comp8316_c0_seq1	-	-	-	n.d.	nodule-enhanced
***RIT/NAP1***	AES90091	comp4244_c0_seq2	*Dgc4066*	1	1	n.d.	similar levels
***PIR1***	XP_003626502	comp3407_c0_seq1	*-*	-	-	n.d.	similar levels
***SYMREM1***	AEX20500	comp279_c0_seq1	*Dgc390, Dgc6526*	8	8	similar levels	nodule-specific
		comp115_c0_seq1	*Dgc955*	8	0	nodule-enhanced	
***NUP133***	CAI64811	comp6865_c0_seq1	*-*	-	-	n.d.	root-enhanced
***IPD3/CYCLOPS***	ABU63668	comp2070_c0_seq4	*-*	-	-	n.d.	nodule-enhanced
***NIN***	CAB61243	comp564_c0_seq1	*Dgc1007*	7	0	nodule-enhanced	nodule-specific
***ERN1***	ABW06102	comp6569_c0_seq1	*-*	-	-	n.d.	similar levels
***NSP1***	ABK35066	comp755_c1_seq1	*Dgc3874*	0	2	n.d.	nodule-enhanced
***NSP2***	XP_003601076	comp1841_c1_seq1	*Dgc1391*	4	2	n.d.	root-specific
***HAP2-1***	ABP68866	comp860_c0_seq1	*-*	-	-	n.d.	nodule-specific
		comp999_c0_seq3	*Dgc4344*	2	0	n.d.	
***CERBERUS/LIN***	C6L7U1	comp2398_c0_seq12	*Dgc5271*	2	2	similar levels	nodule-enhanced
***HK1/CRE1***	XP_003617960	comp11620_c0_seq, comp1838_c0_seq4, comp6496_c0_seq2, comp13545_c0_seq2	*-*	-	-	n.d.	similar levels
***HMGR1***	ABY20972	comp406_c0_seq2	*Dgc1796, Dgc1881, Dgc4998*	5	15	n.d.	root-enhanced
		comp3746_c0_seq1	*Dgc1415*	0	6	n.d.	
***VPY***	ADC33495	comp804_c0_seq1	*Dgc3012*	0	4	nodule-enhanced	nodule-enhanced
***PUB1***	DAA33939	comp11879_c0_seq4	*-*	-	-	root-enhanced	nodule-enhanced
		comp7088_c0_seq2	*Dgc2263*	1	4	n.d.	

qPCRs were performed based on RNA from nodules and roots of non-inoculated plants. The last column refers to the expression in uninfected legume roots *vs*. mature legume nodules. If expression levels in roots and nodules differed by a factor of 2 or more, expression is described as nodule-enhanced or root-enhanced, respectively. If expression levels in both organs differed by a factor of 100 or more, expression is described as root- or nodule-specific, respectively. When more than one *D. glomerata* homolog of a legume gene is listed, the *D. glomerata* contig with the strongest homology between its implicated protein and the legume protein(s) is listed first. Homologies between the legume proteins and the *D. glomerata* proteins are given in Table S2.

n.d. – not determined.

Analysis of the differentially expressed genes showed that a gene encoding a homolog of the nodule-specific transcription factor NIN (nodule inception protein) of *Lotus japonicus*, a central regulator in nodulation responsible for the de-differentiation of root cortical cells and required for nodule initiation [35], [36], was induced in *D. glomerata* nodules compared to roots (*Dgc1007*; Table S1A). In legumes, *NIN* expression is induced by cytokinin signaling, which is induced by Nod factor signaling. The full-size sequence of *Dgc1007* (*DgNIN;* GenBank accession no. JX912727) was obtained, and its nodule-enhanced expression was confirmed by RT-qPCR (Table 3). Interestingly, in *D. glomerata NIN* expression was induced only 7.3 times in nodules compared to roots, i.e., it was not nodule-specific as in legumes (Table 1). Nodule-upregulated *NIN* homologs had also been found in *Alnus glutinosa* and *Casuarina glauca* [19]. Thus, the transcription factor NIN plays a role in all root nodule symbioses; however, it remains to be shown whether in actinorhizal symbioses *NIN* expression is induced by cytokinin signaling like in legume symbioses.

There are some differences in differential expression of the *D. glomerata* homologs in that the genes encoding the closest homologs of *NFR1/LYK3* and of *NSP2* are not expressed root-specifically in *D. glomerata*, which might be explained by a different gene family situation in this species. Instead of recruitment by gene duplication, recruitment by expansion of function might have taken place in the evolution of actinorhizal Cucurbitales. For some homologs (CCaMK/DMI3, CASTOR/POLLUX, NUP133, CYCLOPS, PIR1, ERN1, HAP2-1, CRE1), no data on differential expression in *D. glomerata* are available (Table 3).

In summary, homologs of most components of the common symbiosis pathway known from legumes and of several genes essential for legume nodule formation were found in the *D. glomerata* nodule transcriptome. Furthermore, Table 3 shows that in most cases where the expression profiles of these genes in roots and nodules could be assessed, they are similar to those in legumes. The data support the conclusion of Markmann et al. [33] that the effect of SymRK on nodule induction shows that the common symbiosis pathway is required for the induction of actinorhizal nodules on *D. glomerata* roots, as it is required for the induction of actinorhizal nodules on *C. glauca* (Fagales) [19], [37], [38].

### Homologs of genes encoding proteins involved in infection thread formation are present, but do not always show the same differential expression as in legumes

Two exceptions were found where the expression profile of the *D. glomerata* homolog of a nodulation-related gene did not fit the expression profile of the legume homolog. The function of the corresponding genes (*PUB1, CERBERUS/LIN*) [39], [40] are related to infection thread growth. While infection thread growth mechanisms are similar in legumes and actinorhizal Fagales, they seem to be different in actinorhizal Cucurbitales (reviewed in [3]). In particular, no pre-infection thread structures, which have been described for legumes and *A. glutinosa*, are formed in *D. glomerata* nodules [12], [13], [41]. Furthermore, transcellular infection threads in Cucurbitales do not grow through the cell center as in legumes and actinorhizal Fagales, filling the cell with branching infection threads from the center outward, but remain in the periphery of the cell, filling it from the periphery inward [12]. Thus, in contrast with actinorhizal Fagales and Rosales, infected cells of nodules of Cucurbitales retain a large central vacuole [12]. In this context, it is interesting that homologs of all genes encoding proteins that have been associated with infection thread growth in legumes (VPY, PUB1, CERBERUS/LIN, RIT/NAP1, PIR1, SYMREM1) [39], [40], [42], [43], [44] have been found in the *D. glomerata* nodule transcriptome. In legumes, expression of *VPY, CERBERUS/LIN* and *PUB1* is induced in nodules compared to roots [39], [40], [42]. Upregulation of *CERBERUS/LIN* in nodules was also confirmed for *C. glauca* (homologs of the other genes have not been identified in Fagales yet) [19]. However, the *D. glomerata* homologs of *CERBERUS/LIN* and of *PUB1* (GenBank accession number KC145163) were not upregulated in nodules when analysed using qPCR. Only the *VPY* homolog (GenBank accession number KC145164) was induced in nodules (Tables 1,3). The situation for the symbiotic remorin (SYMREM1; MtREM2.1 in [45]) was more complicated as there were three remorin homologs present in the Trinity assembly and due to the intrinsically high sequence variability of the N-terminal domains it is difficult to determine which is the closest homolog of the symbiotic remorin (data not shown). Phylogenetic analysis using the remorin protein families from *Arabidopsis thaliana* and *M. truncatula* [45] showed that with the inclusion of the three *D. glomerata* nodule remorins, the latter map in a sister clade to the symbiotic remorins of *M. truncatula* (Figure S2). Expression of one of them, DgREM2 (Table S1A), was enhanced in nodules compared to roots as confirmed by qPCR (Tables 1, 3), similar to the symbiotic remorin genes in *M. truncatula* [44], *A. glutinosa* and *C. glauca* [19]. In summary, so far the relative expression levels in roots and nodules of two *D. glomerata* genes (*DgVPY, DgREM2*) the products of which have been implicated in infection thread growth are consistent with those of their legume homologs, while the relative expression levels of two other genes (*DgCERBERUS/LIN, DgPUB1*) are not. Yet, this might be explained by the recruitment of different members of the corresponding gene families in Cucurbitales. Hence, no conclusion can be drawn regarding infection thread growth mechanisms in Cucurbitales *vs.* Fagales/legumes based on gene expression data.

### Genes encoding proteases and cysteine-rich peptides transcribed in roots and nodules of actinorhizal plants

The composition of proteases and cysteine-rich peptides differs between roots and nodules and between *A. glutinosa, C. glauca* and *D. glomerata*. The family of cysteine proteases that is strongly upregulated in *A. glutinosa* nodules [16] has no representatives in either *C. glauca* or *D. glomerata* nodules or roots [19]. However, there are cysteine protease genes expressed at high levels in roots of *A. glutinosa, C. glauca* and *D. glomerata* which are downregulated in nodules; in the case of *A. glutinosa* and *C. glauca*, they are homologs of xylem cysteine protease 1 [19], while in the case of *D. glomerata*, they are papain-type cysteine proteases (Table S1B). Among aspartic proteases, nepenthesin-type proteases are downregulated in *A. glutinosa* as well as in *C. glauca* nodules, but upregulated in *D. glomerata* nodules (Table S1A) [19], while other types of aspartic proteases are upregulated in *C. glauca* nodules, but none is upregulated in *A. glutinosa* nodules. Among serine proteases, a homolog of the nodule-specific subtilisin-type proteases characterized in infection thread-containing cells of *A. glutinosa* and *C. glauca* [17], [46], [47] is also present, and seemingly also nodule-specific, in *D. glomerata* nodules (Table S1A). The expression of cucumisin-type proteases is induced in nodules of *C. glauca* and *D. glomerata,* but not of *A. glutinosa* (Table S1A) [19].

It is striking that cytosolic metallothioneins (MTs) constitute the bulk of cysteine-rich peptides in roots of *D. glomerata* (Table S1B). In principle, this is also the case in *A. glutinosa* and *C. glau*ca, although there *MT* gene expression levels in roots and nodules are more similar [19]. In all three actinorhizal species examined, apoplastic defensins are induced in nodules compared to roots. The full-size sequences of the two nodule-specific cysteine-rich peptide cDNAs representing the genes expressed at high levels in *D. glomerata* nodules (Table S1A), *Dgc156* and *Dgc232,* were obtained (GenBank accession numbers HQ005271 and HQ005272, respectively).

**Figure 1:**
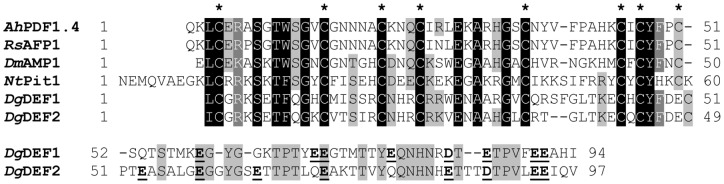
Defensin amino acid sequence alignment. The amino acid sequences of the mature peptides of DgDEF1 and DgDEF2 are compared with the sequences of four mature defensin peptides of the A3 class, a defensin from *Arabidopsis halleri* (AhPDF1.4; GenBank accession no. AY961379.1), the Antifungal Protein 1 from *Raphanus sativus* seeds (RsAFP1) [55], a defensin from *D. merckii* (AMP1) [53] and the aluminum-induced tobacco protein (NtPit1) [52]. Gaps to optimize the alignment were introduced using the program ClustalW (EMBL), and the editor GeneDoc was used to present the alignment [89]. Identical amino acids at conserved positions are labeled by inverse print, whereas positions without full amino acid conservation are shaded in gray. Asterisks mark the cysteine residues conserved in the defensins from plants other than *D. glomerata*. All negatively charged amino acids in the unique C-terminal domains of DgDEF1 and DgDEF1 are marked by bold print and underlined.

*Dgc156*, a 639 bp cDNA, encodes a protein of altogether 120 amino acids with a molecular weight of 13.77 kDa. According to Euk-mPLoc 2.0 (http://www.csbio.sjtu.edu.cn/bioinf/euk-multi-2/)[48], the protein localizes to the apoplast; according to SignalP [49], the first 26 amino acids represent the signal peptide, resulting in a mature protein of 94 amino acids, a molecular weight of 11.44 kDa and an IEP of 5.79. The amino acid sequence shows homology with defensins; accordingly, the protein was termed DgDEF1. Plant defensins are small basic apoplastic proteins of typically 45–55 amino acids and a net positive charge, with eight cysteine residues that form four disulfide bridges [50]. It should be pointed out that the net negative charge and the acidic IEP of DgDEF1 are due to the acidic C-terminal domain comprising 40 amino acids; the N-terminal defensin domain does indeed have a net positive charge and an alkaline IEP (Figure 1).

The eight cysteine residues in the N-terminal domain of the mature DgDEF1 protein form a pattern of C-X_10_-C-X_5_-C-X_3_-C-X_10_-C-X_9_-C-X-C-X_4_-C, which is very similar, though not identical, to the cysteine pattern of class A3 defensins (Figure 1) and group III of defensin-like proteins in grapevine (C-X_5–10_-C-X_4–6_-C-X_3_-C-X_9–15_-C-X_5–12_-C-X-C-X_3_-C) [51]. Among characterized defensins, DgDEF1 shows the highest homology with representatives of class A3, an aluminum-induced tobacco protein [52], a defensin of *Dahlia merckii* [53], [54] and the Antifungal Protein 1 from radish seeds (RsAFP1) [55], [56]. It shows lower homology with the A2 defensin from *Aesculus hippocastanum* [57] and even lower homology with the B2 thionins from wheat and barley [58]. Detailed analysis revealed that *DgDEF1* is a member of a small nodule-specific defensin subfamily including *DgDEF2* (*Dgc845*; Table S1A and Table 1; GenBank accession no. JX912726). DgDEF2 represents a 123 amino acid protein the first 26 amino acids of which represent the signal peptide; as a result, the mature protein consists of 97 amino acids and has a molecular weight of 10.74 kDa and an IEP of 5.87. Thus, both members of this defensin family contain the unusual C-terminal domain resulting in an acidic IEP of the putative mature protein. The cysteine spacing differs between DgDEF1 and DgDEF2 (Figure 1).

While both *A. glutinosa* and *C. glauca* contain gene families of defensin-like peptides the expression of which is highly induced in nodules compared to roots [19] (Figure S3), no defensin from either species contains the unusual C-terminal domain found in DgDEF1 and DgDEF2. Nevertheless, apart from this feature, the nodule-specific or highly nodule-enhanced defensin-like peptides from *A. glutinosa* and *C. glauca* belong to the same group of defensins as DgDEF1 and DgDEF2 (Figure S3).

*Dgc232,* a 645 bp cDNA, encodes a small basic cysteine-rich peptide (CRP) of which no homologs exist in nodules of *A. glutinosa* and *C. glauca* or in the databases at www.ncbi.nlm.nih.gov; the gene was termed *DgCRP1.* The encoded protein has a molecular weight of 12.38 kDa and consists of 115 amino acids. According to Euk-mPLoc 2.0, it is targeted to the apoplast, with a signal peptide (SignalP) of 29 amino acids, resulting in a mature protein of 86 amino acids, a molecular weight of 9.37 kDa and an IEP of 8.36. DgCRP1 contains seven cysteine residues, one near the N-terminus and the other six in the C-terminal domain, the latter forming a pattern of C–X_3_–C–X_4_–C–X_10_–C–X_3_–C–X_3_–C. So far, this pattern has not been found in small cysteine-rich proteins from plants [59].

Eukaryotes produce small cysteine-rich antimicrobial peptides (CRPs) as an innate defense against pathogens [59]. Defensins, a large group of those peptides, induce the permeabilization of fungal membranes [60], [61], [62]. However, the function of CRPs is not restricted to defense. Legumes have been shown to contain large gene families encoding different groups of CRPs [63], and a group of nodule-specific cysteine-rich peptides (NCRs) has been found to control bacterial differentiation including the amplification of the rhizobial genome in nodules of certain legumes [64]. In actinorhizal symbioses, the plant affects bacterial differentiation as exemplified by the fact that shape and subcellular position of *Frankia* vesicles formed *in planta* are host-specific [3], though no data on genome amplification of *Frankia* in symbiosis are available. It is tempting to speculate that not only legumes but also actinorhizal plants control the differentiation of their bacterial endosymbionts by cysteine-rich peptides. However, CRPs have also been found to be involved in developmental processes [65], [66], for instance in guiding pollen tube growth [67], [68]. Since both pollen tubes and infection threads employ the tip growth mechanism, this might also offer a function for nodule-specific defensins and other cysteine-rich peptides in controlling the infection process.

### Thiamine biosynthesis is upregulated in actinorhizal nodules

Nodule-enhanced genes encoding enzymes involved in thiamine biosynthesis (*Dgc1186, Dgc1757, Dgc1813, Dgc2133, Dgc1072* and *Dgc1622,* respectively; Table S1A) show a parallel with actinorhizal nodules from *A. glutinosa,* where a gene encoding AgThi1 was strongly induced in nodules compared to roots [69]. This was also observed for the *AgThi1* homolog in *C. glauca* [19]. No similar induction of thiamine biosynthesis genes has been reported for legume nodules. Thus, actinorhizal plants from two different phylogenetic subgroups seem to induce thiamine biosynthesis in nodules, while legumes do not. Do actinorhizal plants provide thiamine to their microsymbionts in symbiosis? At least in the microsymbiont of *A. glutinosa,* ACN14a, thiamine biosynthesis, as indicated by the expression levels of *ThiC,* is not significantly downregulated in symbiosis compared to N-replete conditions in the free-living state, nor is it induced during free-living nitrogen fixation [70].

## Conclusions

The identification of many components of the common symbiosis signal transduction pathway in *D. glomerata* nodules opens possibilities for detailed comparisons between root nodule symbiosis of legumes, actinorhizal Fagales and actinorhizal Cucurbitales. The mechanisms of induction of organogenesis and internalization of the microsymbionts in nodule cells can be assessed, at least with regard to the question after the conservation of the mechanisms identified for legumes. A certain subgroup of nodule-specific defensin-like peptides which do not appear in legume nodules has now been found in transcriptomes of actinorhizal plants from different phylogenetic subgroups indicating that these peptides might play a role specific to actinorhizal symbioses. Which role defensin-like peptides play and why the *D. glomerata* representatives contain an acidic C-terminal domain remains to be examined.

## Materials and Methods

### Plant material

*Datisca glomerata* (Presl.) Baill seeds were originally obtained from plants in Vaca Hills, California. No specific permissions were required because the collection took place from plants growing in a stream-bed, which was not privately owned. *D. glomerata* is not an endangered or protected species in any part of its geographical range, by either state or federal law. Plants were grown in a greenhouse and watered with 1/4 strength Hoagland's [71] once per week and twice per week with deionized water. Light conditions in the greenhouse were 150–300 μmol photons m^−2^ s^−1^; temperature set points were 22°C/19°C at 13 h light/11 h dark. Seeds were germinated on germination soil (Weibull Trädgard AB, Hammenhög, Sweden). When the plants had reached a height of about 20 cm, they were transferred to pots containing sand (0–2 mm Quartz; Rådasand AB, Lidköping, Sweden) and soil from nodulated *D. glomerata* plants containing spores of *Candidatus* Frankia datiscae Dg1, a non-cultured *Frankia* strain originating from *Coriaria nepalensis* nodules from Pakistan [72]. For transcriptome analysis, nodules and roots were harvested four to six weeks after transfer. Nodules with one or two lobes were considered young, and noduels composed of more than two lobes were considered mature. Roots from nodulated plants were cut off ca. 4 cm above the root tip. For RT-qPCR analysis, plants were grown on germination soil throughout.

### Preparation of cDNA from roots and nodules of *D. glomerata* for transcriptome analysis

The RNA isolation protocol used was modified after Chomczynski [73]. Plant tissue ground in liquid N_2_ and then transferred to a 2 ml microcentrifuge tube containing 1 ml of pre-warmed extraction buffer (65°C, 2% CTAB, 2% PVP (K30), 100 mM Tris-HCl pH 8.0, 25 mM EDTA, 2 M NaCl, 0.5 g/l spermidine, 2% ß-mercaptoethanol) per 200 mg of plant material and mixed by inverting and vortexing. The mixture was subjected to RNA extraction twice with an equal volume of (25∶24∶1) phenol:chloroform:isoamyl alcohol and once with (24∶1) chloroform:isoamyl alcohol. Separation of phases was achieved by centrifugation at 10,000×*g* at room temperature for 10 min. The resulting RNA was precipitated using 1/10 volume of 3 M sodium acetate pH 5.2 and 2.5 volumes of absolute ethanol at −20°C overnight. The RNA from *D. glomerata* roots was labeled ‘DgR’, from young nodules ‘DgNy’ and from mature nodules ‘DgNm’.

Poly(A) RNA was prepared from RNAs DgR, DgNy and DgNm by Eurofins MWG Operon (Ebergsberg, Germany). Prior to purification, nodule RNA samples were mixed (DgNy/DgNm 1∶2). The poly(A) RNA was used to synthesize double-stranded cDNA [74] using a specific oligo(dT) adapter primer, which carried the 454 adapter B sequence. Then, the cDNA was cut with the restriction enzyme *Nla*III. Subsequently, the purified *Nla*III fragments were ligated to a short double-stranded adaptor which carried the 454 adapter A sequence. Finally, the distal 3′-cDNA fragments which carried the poly(A) tails were specifically PCR-amplified to about 40 ng/μl (for cycle numbers see Table S3). The barcode sequences which were attached to the 5′-ends of the cDNAs are included in Table S3.

### Massive parallel sequencing and sequence evaluation

Contig assembly and calculation of occurrences of a particular contig in the nodule library *vs.* root library was performed by Eurofins MWG Operon. Sequence homologies were analysed using BlastX at www.ncbi.nlm.nih.gov. The statistical significance of differences in transcripts levels between roots and nodules was analysed by calculation of the R parameter using modified Poisson statistics as described by Journet et al. [27]. For every contig, the probability was calculated for two hypotheses. H_0_: number of copies is equal in both libraries. H_1_: one library contains more copies than the other. The ratio (R) between the probabilities H_1_/H_0_ was used to decide whether the difference of copy number between nodule and root libraries was significant. A difference between expression levels was considered significant when R>10.

Protein sequence analysis was performed using the GCG program package (Wisconsin Genetics Computer Group) and the I-TASSER platform [75].

### Illumina paired-end sequencing and sequence evaluation

Nodule RNA was isolated as described before and prepared for sequencing using the TruSeq cDNA preparation kit (Illumina, San Diego, CA, USA). Paired-end sequencing to 100 bp was performed on an Illumina HiSeq2000 instrument using v. 1.5 flow cells, resulting in about 132 million paired-end reads.

Reads were assembled using Trinity version 2011-10-29 [28]. CAP3 [29] was then used to perform a ”meta-assembly” where the contigs of the Trinity assembly were assembled together with the contigs obtained from 454 sequencing. Default parameters were used for both programs.

### Amplification of full-size cDNAs

Total RNA was isolated from *D. glomerata* nodules using RNeasy Plant Mini Kit (Qiagen, Hilden, Germany). To obtain the full-size cDNA sequences, 5′- and 3′-rapid amplification of cDNA ends (RACE) was performed. Reverse transcription was performed on 1 µg of total RNA using MuLV RT (Fermentas, St. Leon-Rot, Germany) and 5′-CDS primer A together with the SMART II oligo for 5′-RACE-Ready cDNA, or 3′-CDS primer A (Clontech, Mountain View, CA, USA) for 3′-RACE-Ready cDNA, respectively, in a final volume of 20 µl following recommendations of the manufacturer. RACEs were performed according to the SMART^TM^ RACE cDNA Amplification protocol (Clontech) on 2.5 µl aliquots of the first-strand cDNA, diluted 1∶13 with Tricine-EDTA buffer, with Universal Primer A Mix (Clontech) and the first gene-specific primer (Table S4). The diluted product of the primary PCR was used in the secondary PCR with Nested Universal Primer A (Clontech) and the second gene-specific primer (Table S4). Full-length cDNAs were generated by long distance PCR using 2.5 µl of 5′-RACE-Ready cDNA as template. The PCR program used was 35 cycles of 94°C for 30 s, annealing at temperature 5°C lower than the melting temperature of the primer pair for 30 s and 72°C for 3 min. The PCR products were cloned in pCR2.1-TOPO (5′-RACE products for *DgMnSOD1* (*Dgc73*) and *DgDCAT1* (*Dgc108*); Invitrogen, Carlsbad, CA, USA) or pJET1.2 (all other PCR products; Fermentas) and sequenced.

### Reverse transcription and quantitative real-time PCR (qPCR)

Total RNA was isolated from *D. glomerata* nodules and from roots of non-inoculated plants, using the RNeasy Plant Mini Kit with on-column DNase digestion (Qiagen, Hilden, Germany). Reverse transcription was performed on 2.3 µg total RNA with *Not*I-d(T)_18_ primers in a final volume of 33 µl, using the First-Strand cDNA Synthesis Kit (GE Healthcare AB, Stockholm, Sweden) according to the protocol provided by the manufacturer. All qPCR assays contained 1X Maxima SYBR Green qPCR Master Mix (Fermentas, Vilnius, Lithuania), 325 nM of each primer, 5 µl of diluted cDNA in a total reaction volume of 20 µl. qPCR was conducted on a LightCycler480 (Roche, Mannheim, Germany) under the conditions of 10 min of initial denaturation at 94°C, 40 cycles of 15 sec at 94°C and 30 sec at 60°C followed by a melt curve analysis. Assays were analysed in triplicate with standard curve method [76]. PCR efficiency was calculated in LightCycler480 software with data obtained from the exponential phase of each amplification plot. The transcript data were normalized against the constitutively expressed *D. glomerata* ubiquitin gene (*Dgc205;* Table S5). Primer sequences used in the transcript analysis (Table S5) were designed using the software Primer 3 v. 0.4.0 (http://frodo.wi.mit.edu/primer3/). Data pre-processing, normalization and t-test (p<0.05) were performed with GenEx (version 5.4.1, MultiD Analyses, Göteborg, Sweden).

## Supporting Information

Figure S1 **Preparation of the libraries for sequencing, based on Eveland et al.** [**26**] **.**
(PPTX)

Figure S2 **Phylogenetic tree of the** ***Arabidopsis*** **and** ***Medicago truncatula*** **remorin protein families** [**45**] **and the three remorins from the** ***Datisca glomerata*** **nodule transcriptome (arrows).** The sequences were aligned using ClustalW [90]. The phylogenetic trees were estimated by neighbor-joining analysis using the software PAUP* 4.0b10 (PPC/Altivec) for Macintosh [91]. Bootstrap analysis with 1000 bootstrap replications using the neighborjoining search option of the program PAUP* 4.0b10 was carried out to test the robustness of the internal branches. A remorin from the liver moss *Physcomitrella patens* (GenBank accession no. XP_001752001) served as outgroup.
(PPTX)

Figure S3 **Alignment of the amino acid sequences of DgDEF1 and DgDEF2 with the defensin-like peptides from** ***Alnus glutinosa*** **and** ***Casuarina glauca*** **that show nodule-specific or strongly nodule-enhanced expression: AgDEF1, FQ334620; AgDEF2, FQ344001; AgDEF3, FQ334074; CgDEF1, FQ318729; CgDEF2, FQ362615; CgDEF3, FQ363112; CgDEF4, FQ363205; CgDEF5, FQ320471** [**19**] **.** Gaps to optimize the alignment were introduced using the program ClustalW (EMBL), and the editor GeneDoc was used to present the alignment [89]. Identical amino acids at conserved positions are labeled by inverse print, whereas chemically similar amino acids are shaded in gray. Asterisks label every 10^th^ amino acid position. Putative signal peptides are underlined. Sequence AgDEF1 is clearly truncated at the 5′-end, and sequences AgDEF2 and CgDEF3 most probably are truncated since the encoded proteins lack a signal peptide.
(DOCX)

Table S1 **A.** *Datisca glomerata* genes upregulated in nodules compared to roots. Genes mentioned in the manuscript and not published earlier are given in bold print. **B.** *Datisca glomerata* genes upregulated in roots compared to nodules. Genes mentioned in the manuscript and not published before are given in bold print.
(DOCX)

Table S2 **Homology between legume proteins (GenBank accession numbers given) involved in nodule induction, and the corresponding proteins encoded by** ***Datisca glomerata*** **nodule contigs.** The *Lotus japonicus* (Lj) and/or *Medicago truncatula* (Mt) protein sequences were used for a tBlastN search on the different assemblies of the *D. glomerata* nodule transcriptome. The homology values (E values) for the contigs given in Table 3 are listed. Homologies for *D. glomerata* SYMRK/DMI2 are not given as this gene was already functionally characterized [33].
(DOC)

Table S3 **Preparation of cDNA libraries (1, root cDNA library; 2, nodule cDNA library).** PCR cycles used for cDNA amplification and barcode sequences attached to 5′-ends of cDNAs
(DOCX)

Table S4 **Primers used for amplification of cDNAs from** ***Datisca glomerata.***
(DOCX)

Table S5 **Primers used in quantitative real-time PCR.**
(DOCX)
