# Supplementary material for: Comparison of the Nodule vs. Root Transcriptome of the Actinorhizal Plant Datisca glomerata: Actinorhizal Nodules Contain a Specific Class of Defensins
Source: PLoS One. 2013 Aug 29;8(8):e72442. doi: 10.1371/journal.pone.0072442 (PMC3756986; doi:10.1371/journal.pone.0072442)
Supplement: Figure S3 — Alignment of the amino acid sequences of DgDEF1 and DgDEF2 with the defensin-like peptides from Alnus glutinosa and Casuarina glauca that show nodule-specific or strongly nodule-enhanced expression: AgDEF1, FQ334620; AgDEF2, FQ344001; AgDEF3, FQ334074; CgDEF1, FQ318729; CgDEF2, FQ362615; CgDEF3, FQ363112; CgDEF4, FQ363205; CgDEF5, FQ320471 [19] . Gaps to optimize the alignment were introduced using the program ClustalW (EMBL), and the editor GeneDoc was used to present the alignment [89]. Identical amino acids at conserved positions are labeled by inverse print, whereas chemically similar amino acids are shaded in gray. Asterisks label every 10th amino acid position. Putative signal peptides are underlined. Sequence AgDEF1 is clearly truncated at the 5′-end, and sequences AgDEF2 and CgDEF3 most probably are truncated since the encoded proteins lack a signal peptide. (DOCX) [file pone.0072442.s003.docx]

**Figure S3** Alignment of the amino acid sequences of DgDEF1 and DgDEF2 with the defensin-like peptides from *Alnus glutinosa* and *Casuarina glauca* that show nodule-specific or strongly nodule-enhanced expression: AgDEF1, FQ334620; AgDEF2, FQ344001; AgDEF3, FQ334074; CgDEF1, FQ318729; CgDEF2, FQ362615; CgDEF3, FQ363112; CgDEF4, FQ363205; CgDEF5, FQ320471 [1]. Gaps to optimize the alignment were introduced using the program ClustalW [2], and the editor GeneDoc was used to present the alignment [3]. Identical amino acids at conserved positions are labeled by inverse print, whereas chemically similar amino acids are shaded in gray. Asterisks label every 10^th^ amino acid position. Putative signal peptides are underlined. Sequence AgDEF1 is clearly truncated at the 5’-end, and sequences AgDEF2 and CgDEF3 most probably are truncated since the encoded proteins lack a signal peptide.

* * * * * *
**CgDEF1** 1 MAVLSSFFRILFVVLTFSAVVFAIIPVGAEENPASDPGGWSICTILSKTWHKRC--WETK 58
**AgDEF3** 1 MSVSSFIGVLVIFALVFVFVSPTLPLYR-PTPLPAVINMQPCESLSKTWHGRC--RSTR 56
**AgDEF2** 1 MATAQYLLCGRRSRTWSGFCSYFSSD 26
**CgDEF4**  1 MNKCVRSVNCLAVLVVFIFLVNTVS----SEKGKYDFRKLKSRTWSGPC--FNNR 49
**CgDEF2** 1 MNKSVRSVNYFAVLVIFILLVNTVVYIMVVSSTELELRKFKSETWSGPC--FNIR 53
**CgDEF5** 1 MNKSVRLVNYFAVLVIFILLVNTVS------SQEEKFQKVKSRTWSGPC--FNGR 47
**CgDEF3** 1 MVVSS-QEEFQKVKSRTWSGPC--FNSR 25
**DgDEF1** 1 MANIPKYISLFSIFITLLLLLNEATALCGRKSETFQGHC--MISS 43
**DgDEF2**  1 MANTPKDIPLFAIFITFLLLLNMASAICGRKSETFQGKC--VTSI 43
**AgDEF1** 1 KQCSEGFNRRRSKTWSGPC--FFDS 23


 * * * * * *
**CgDEF1**  59 HCDKHCRKREQADHGACHG--FWHSRCYCYYI---------------------------- 88
**AgDEF3**  57 NCDRHCRNQEKADNGACHG--FWHKKCYCYTSEMCLNTRR-------------------- 94
**AgDEF2** 27 GCDRQCQHWEDAFSGACHG--FPHHACYCYYLCWNTNRIN-------------------- 64
**CgDEF4** 50 GCNNQCQRWEDADYGRCRR--T-LFTCFCYFVIPP------------------------- 81
**CgDEF2** 54 GCYDQCVMWEDADYGHCR-----WFVCYCYFLLPVEEEVQV------------------- 89
**CgDEF5** 48 GCDNQCKEREDADYGGCP-----WFVCYCYFFIVPP------------------------ 78
**CgDEF3**  26 GCDKQCREWEDADYGGCP-----WFVCYCYFLIPPPE----------------------- 57
**DgDEF1**  44 RCNHRCRRWENAARGVCQRSFGLTKECHCYFDECSQTSTMK---EGYGGKTPTYEEGTMT 100
**DgDEF2** 18 RCNHRCRKVENAAHGLCR--TGLTKECQCYFDECPTEASALGEGGYGSETTPTLQEAKTT 101
**AgDEF1**  24 KCDRQCREWEKAEYGDCR---GSFFACSCFFKATYQKVRNHN 62


 * *
**DgDEF1** 101 TYEQNHNRDT--ETPVFEEAHI 120
**DgDEF2** 112 VYQQNHNHETTTDTPVLEEIQV 123

**References**

1. Hocher V, Alloisio N, Auguy F, Fournier P, Doumas P, Pujic P, Gherbi H, Queiroux C, Da Silva C, Wincker P, Normand P, Bogusz D (2011) Transcriptomics of actinorhizal symbioses reveals homologs of the whole common symbiotic signaling cascade. Plant Physiol 156: 700-711.
2. Thompson JD, Gibson TJ, Plewniak F, Jeanmougin F, Higgins DG (1997) The CLUSTAL_X windows interface: flexible strategies for multiple sequence alignment aided by quality analysis tools. Nucl Acids Res 25: 4876-4882.
3. Nicholas K, Nichola HB Jr, Deerfield DW II (1997) GeneDoc: analysis and visualization of genetic variation. EMBnet NEWS 4: 14.
