# Supplementary material for: Comparison of the Nodule vs. Root Transcriptome of the Actinorhizal Plant Datisca glomerata: Actinorhizal Nodules Contain a Specific Class of Defensins
Source: PLoS One. 2013 Aug 29;8(8):e72442. doi: 10.1371/journal.pone.0072442 (PMC3756986; doi:10.1371/journal.pone.0072442)
Supplement: Table S1 — A. Datisca glomerata genes upregulated in nodules compared to roots. Genes mentioned in the manuscript and not published earlier are given in bold print. B. Datisca glomerata genes upregulated in roots compared to nodules. Genes mentioned in the manuscript and not published before are given in bold print. (DOCX) [file pone.0072442.s004.docx]

**Table S1A.** *Datisca glomerata* genes upregulated in nodules compared to roots. Genes mentioned in the manuscript and not published before are given in bold print.

| **454 contig/supercontig** | **Reads in nodules** | **Reads in roots** | **R value** | **Description of encoded protein** |
| --- | --- | --- | --- | --- |
|  |  |  |  |  |
| Dgc217, Dgc931, Dgc1439, Dgc5848, Dgc5727, Dgc2411, Dgc788, Dgs469, Dgc217, Dgc2197, Dgc2868, Dgs1525, Dgc395, Dgs646, Dgc436, Dgc610, Dgs709 | 201 | 2 | 3.54E+040 | *D. glomerata* mRNA for pathogenesis-related protein PR10A [1] |
| Dgc11, Dgc3607, Dgc306, Dgc3298 | 103 | 0 | 3.47E+022 | *D. glomerata* nodule-specific protein Dg93 [2] |
| **Dgc156** | 88 | 0 | 1.66E+019 | **defensin DgDEF1** |
| Dgc15 | 60 | 0 | 1.06E+013 | Citrus blight-associated protein P12 precursor, γ-expansin, natriuretic peptide |
| Dgc4, Dgs49, Dgc962 | 131 | 21 | 4.55E+012 | flavonol synthase |
| **Dgc124, Dgc5071** | 54 | 0 | 4.99E+011 | ***A. glutinosa* Ag12, C*.glauca* Cg12, subtilisin-like protease** [3] |
| Dgc371, Dgc484, Dgc2020, Dgc2181, Dgc3364, Dgc1744, Dgc484 | 53 | 0 | 3.00E+011 | peroxidase |
| Dgc22, Dgc181, Dgc3184, Dgc825, Dgs863, Dgs1246, Dgc1487, Dgc6606, Dgc2629 | 68 | 3 | 1.26E+011 | fluG; nodulin / glutamate-ammonia ligase-like protein |
| Dgc271 | 49 | 0 | 3.91E+10 | plastidic fructose bisphosphate aldolase (ALDP) |
| Dgc487, Dgc2962, Dgc2953, Dgc1858, Dgc1468, Dgc5351, Dgs2003, Dgc576 | 77 | 7 | 1.04E+10 | elicitor-responsive protein 3 |
| Dgc179, Dgc723 | 45 | 0 | 5.09E+09 | Citrus blight-associated protein P12 precursor, gamma-expansin, natriuretic peptide |
| Dgc40, Dgc951, Dgs1218 | 41 | 0 | 6.64E+08 | phosphoribulokinase, chloroplast precursor |
| **Dgc232, Dgc298** | 38 | 0 | 1.44E+08 | **cysteine-rich protein DgCRP1** |
| Dgc2, Dgc14, Dgc2217 | 121 | 30 | 1.28E+08 | *D. glomerata* glutamine synthetase 2 (DgGS1-1) [4] |
| Dgc379, Dgc410 | 36 | 0 | 5.19E+07 | hypoxia induced protein conserved region containing protein |
| Dgc63, Dgc68, Dgc2271 | 57 | 6 | 8.29E+06 | major latex protein homologue (MLP1) [1] |
| Dgc108 | 24 | 0 | 1.20E+05 | dicarboxylate transporter DgDCAT1, homolog of AgDCAT1 [5] |
| Dgc133 | 24 | 0 | 1.15E+05 | phosphoglucomutase/phosphomannomutase C terminal; thioesterase superfamily |
| Dgc1188, Dgc1403, Dgc4203 | 23 | 0 | 6.90E+04 | alanine:glyoxylate aminotransferase, peroxisomal |
| Dgc362 | 22 | 0 | 4.15E+04 | nijmegen breakage syndrome 1 protein (nibrin) |
| **Dgc1186, Dgc1757, Dgc1813, Dgc2133, Dgc1072** | 21 | 0 | 2.49E+04 | **thiamine biosynthesis (thiC) family protein** |
| Dgc158, Dgc657 Dgc1078 Dgc404 | 36 | 4 | 1.64E+04 | calreticulin |
| Dgc92, Dgc695 Dgc2178 Dgc2243 | 36 | 4 | 1.64E+04 | aldose 1-epimerase |
| Dgc208 | 20 | 0 | 1.50E+04 | cysteine-rich repeat secretory protein 38-like (salt stress response/antifungal) |
| Dgc768 | 20 | 0 | 1.50E+04 | protein with unknown function, one transmembrane domain |
| Dgc182 | 20 | 0 | 1.50E+04 | WAK2 (wall-associated kinase 2); protein serine/threonine kinase |
| Dgc305, Dgc640, Dgc1224 | 20 | 0 | 1.50E+04 | 60S ribosomal protein L37 |
| Dgc453, Dgc1846 | 19 | 0 | 9.00E+03 | wall-associated receptor kinase 2-like |
| Dgc26, Dgs172 | 45 | 9 | 4.05E+03 | snakin (anti-microbial peptide) |
| Dgc444 | 17 | 0 | 3.25E+03 | oxidoreductase Tas aldo/keto reductase family |
| Dgc789 | 28 | 3 | 2.04E+03 | methylthioribose-1-phosphate isomerase |
| Dgc994 | 16 | 0 | 1.95E+03 | heavy metal transport/detoxification domain-containing protein |
| Dgc758, Dgc2198, Dgc2259, Dgc970, Dgc2490, Dgc6376 | 51 | 13 | 1.78E+03 | glyceraldehyde-3-phosphate dehydrogenase |
| Dgc1060, Dgc5675, Dgc2609 | 33 | 5 | 1.67E+03 | 60S ribosomal protein L7a |
| Dgc53, Dgc300, Dgs494 | 23 | 2 | 919.37 | carbonic anhydrase |
| Dgc174 | 19 | 1 | 751.99 | pyruvate dehydrogenase E1 β subunit |
| Dgc227, Dgc747, Dgc1469, Dgc6238 | 19 | 1 | 751.99 | α chain of nascent polypeptide associated complex |
| Dgc983, Dgc1552, Dgc1410, Dgc2449, Dgc2236, Dgc4372 | 19 | 1 | 751.99 | enolase (2-phospho-D-glycerate hydrolase) |
| Dgc485 | 14 | 0 | 704.32 | serine carboxypeptidase |
| **Dgc374** | 14 | 0 | 704.32 | **cucumisin-like subtilisin family protease** |
| Dgc462, Dgc1855, Dgc1295 | 12 | 42 | 484.71 | glutathione S-transferase |
| Dgc1025 | 13 | 0 | 423.16 | cysteine-rich repeat secretory protein |
| Dgc848, Dgc2578, Dgc3284, Dgc4574 | 21 | 2 | 390.45 | prunasin hydrolase, β-glucosidase |
| Dgc2206, Dgc2563 | 17 | 1 | 300.88 | zinc finger CCCH domain-containing protein 53-like |
| Dgc532, Dgc847 | 17 | 1 | 300.88 | mitochondrial 2-oxoglutarate/malate carrier protein |
| Dgc918, Dgc3319, Dgc1045, Dgc2845 | 28 | 5 | 259.33 | eukaryotic translation initiation factor 2, protein translation factor SUI1-1 |
| Dgc491, Dgc796, Dgc2187, Dgc1659, Dgc6627, Dgc1923 | 20 | 2 | 255.80 | glucose-6-phosphate/phosphate translocator 2, chloroplastic |
| Dgc587, Dgc5171 | 12 | 0 | 254.24 | CCAAT-binding transcription factor subunit A |
| Dgc1834 | 12 | 0 | 254.24 | calcium-binding EF-hand-containing protein (Ca2+ sensor or signal modulator) |
| Dgc1228 | 32 | 7 | 215.83 | plastidic 23S rRNA |
| Dgc350 | 16 | 1 | 191.13 | *D. glomerata* mRNA for 2-on-2 hemoglobin (*glb3* gene) [6] |
| Dgc148 | 22 | 3 | 180.06 | glutamate decarboxylase |
| Dgc1132, Dgc4564 | 11 | 0 | 152.75 | annexin |
| Dgc551 | 11 | 0 | 152.75 | phosphoenolpyruvate-carboxylase kinase |
| Dgc73 | 24 | 4 | 145.14 | Mn-superoxide dismutase II |
| Dgc428 | 21 | 3 | 121.84 | chloroplast ferritin 2 precursor |
| Dgc319, Dgs869, Dgs2954 | 15 | 1 | 121.82 | 60S ribosomal protein L7 |
| Dgc1637 | 10 | 0 | 91.77 | AMP-activated protein kinase, g regulatory subunit, sucrose nonfermenting protein 4 |
| Dgc1092 | 10 | 0 | 91.77 | PsbM protein (from photosystem II) |
| Dgc522 | 10 | 0 | 91.77 | ubiquitin extension protein |
| Dgc1289 | 10 | 0 | 91.77 | 2-C-methyl-D-erythritol 2,4-cyclodiphosphate synthase (in the non-mevalonate deoxyxylulose (DOXP) pathway for isoprenoid biosynthesis) |
| Dgc3334, Dgc5838 | 10 | 0 | 91.77 | α-L-arabinofuranosidase |
| Dgc873, Dgc921 | 10 | 0 | 91.77 | glucan endo-1,3-β-glucosidase-like protein |
| Dgc1763, Dgc5118, Dgc6499 | 10 | 0 | 91.77 | lysosomal β-D-glucosidase |
| Dgc2239, Dgc5192, Dgc5324, Dgc2704 | 14 | 1 | 77.93 | chaperone protein DnaJ |
| Dgc164 | 3 | 18 | 59.15 | calmodulin |
| Dgc314, Dgc3197, Dgc5625, Dgc683, Dgc840, Dgc2246 | 93 | 48 | 55.76 | cation/calcium exchanger-like protein (weak homology) |
| Dgc2482, Dgc6629, Dgc5852 | 9 | 0 | 55.14 | annexin |
| Dgc808 | 9 | 0 | 55.14 | pathogenesis-related protein PR10A (homologous, not identical) [1] |
| Dgc608 | 9 | 0 | 55.14 | acyl-CoA thioesterase |
| Dgc3203 | 9 | 0 | 55.14 | auxin-induced protein PCNT115, aldo/keto reductase |
| Dgc1723, Dgc6816 | 9 | 0 | 55.14 | tubulin β-chain |
| Dgc915, Dgc1620 | 9 | 0 | 55.14 | conserved protein membrane protein YMR155W-like |
| Dgc1752, Dgc3787 | 9 | 0 | 55.14 | fumarylacetoacetate hydrolase |
| Dgc1438, Dgc2010, Dgc4952 | 9 | 0 | 55.14 | pyruvate decarboxylase |
| Dgc1544, Dgc2547, Dgc4234 | 9 | 0 | 55.14 | serpin (serine proteinase inhibitor) |
| Dgc1176 | 13 | 1 | 50.07 | neutral/alkaline invertase |
| Dgc326, Dgc674 | 16 | 2 | 49.22 | alanine-glyoxylate transaminase, mitochondrial |
| Dgc135 | 16 | 2 | 49.22 | biotin carboxyl carrier protein of acetyl-CoA carboxylase |
| Dgc832, Dgc1271, Dgc2830, Dgc3064 | 27 | 7 | 42.89 | 60S ribosomal protein L19 |
| Dgc21 | 34 | 11 | 36.80 | 60S ribosomal protein L10a |
| Dgc79 | 5 | 21 | 36.33 | mitochondrial phosphate transporter |
| Dgc706, Dgc2328 | 8 | 0 | 33.13 | dehydroascorbate reductase class glutathione transferase DHAR2 |
| Dgc1520 | 8 | 0 | 33.13 | thiosulfate sulfurtransferase |
| Dgc2297 | 8 | 0 | 33.13 | heat shock protein |
| **Dgc955** | 8 | 0 | 33.13 | **Remorin DgREM2** |
| Dgc2521 | 8 | 0 | 33.13 | sucrose nonfermenting 4-like protein, AMP-activated protein kinase |
| **Dgc1622** | 8 | 0 | 33.13 | **thiamine thiazole synthase, chloroplastic** |
| Dgc1005 | 8 | 0 | 33.13 | potassium-sodium symporter HKT1 |
| Dgc1239 | 8 | 0 | 33.13 | pectate lyase |
| Dgc1108 | 8 | 0 | 33.13 | heavy-metal-associated domain-containing protein |
| Dgc1568 | 8 | 0 | 33.13 | protein arginine n-methyltransferase 1 |
| Dgc944 | 8 | 0 | 33.13 | receptor protein kinase CLAVATA1 precursor |
| Dgc2991 | 8 | 0 | 33.13 | BRASSINOSTEROID INSENSITIVE 1-associated receptor kinase 1 precursor, LRR receptor-like serine/threonine-protein kinase FEI 1-like precursor |
| Dgc790, Dgc4967 | 8 | 0 | 33.13 | polygalacturonase |
| **Dgc1227, Dgc2304** | 8 | 0 | 33.13 | **aspartic proteinase nepenthesin-2** |
| Dgc1478, Dgc2222 Dgc2589 | 8 | 0 | 33.13 | RNase |
| Dgc1305, Dgc1122 | 15 | 2 | 33.03 | syntaxin |
| Dgc388 | 12 | 1 | 32.32 | 60S ribosomal protein L39 |
| Dgc570, Dgc1362, Dgc3321, Dgc3068, Dgc1476, Dgc2015 | 29 | 9 | 25.15 | 40S ribosomal protein S29 |
| Dgc165, Dgc3627 | 21 | 5 | 23.27 | protein disulfide isomerase family protein |
| Dgc1044, Dgc1614 | 14 | 2 | 22.32 | glucose-1-phosphate adenylyltransferase large subunit, chloroplastic/amyloplastic |
| Dgc1656, Dgc4019, Dgc5508, Dgc3028 | 14 | 2 | 22.32 | acyl carrier protein, plastidic |
| Dgc2690, Dgc4394 | 11 | 1 | 20.98 | 6,7-dimethyl-8-ribityllumazine synthase |
| Dgc2766, Dgc3095 | 7 | 0 | 19.90 | CDC27/NUC2-like protein |
| Dgc3281, Dgc6143 | 7 | 0 | 19.90 | cysteine-rich repeat secretory protein 38-like |
| Dgc1148, Dgc6670 | 7 | 0 | 19.90 | anther-specific protein SF2, defensin-like protein, g-thionin |
| Dgc3198, Dgc6004 | 7 | 0 | 19.90 | NADH dehydrogenase [ubiquinone] iron-sulfur protein 5-B-like |
| Dgc1951, Dgc2237 | 7 | 0 | 19.90 | ribonuclease T2 family protein |
| Dgc1098 | 7 | 0 | 19.90 | 40S ribosomal protein S29 |
| Dgc1075 | 7 | 0 | 19.90 | phosphatidylinositol 4-kinase PI4K |
| **Dgc845** | 7 | 0 | 19.90 | **DgDEF2, defensin-like protein, high homology with DgDEF1** |
| Dgc969 | 7 | 0 | 19.90 | serine decarboxylase, histidine decarboxylase, aromatic amino acid decarboxylase |
| Dgc1100 | 7 | 0 | 19.90 | zinc finger CCCH domain-containing protein, RNA M5U methyltransferase |
| Dgc3678 | 7 | 0 | 19.90 | plastid developmental protein DAG |
| Dgc958 | 7 | 0 | 19.90 | heavy-metal-associated domain-containing protein |
| Dgc1244 | 7 | 0 | 19.90 | 60S ribosomal protein L7-4 |
| Dgc998 | 7 | 0 | 19.90 | shaggy-related protein kinase epsilon, glycogen synthase kinase 3 |
| Dgc888 | 7 | 0 | 19.90 | deoxyhypusine synthase |
| **Dgc1007** | 7 | 0 | 19.90 | **nodule inception protein (NIN)** |
| Dgc1689, Dgc3134, Dgc5847 | 7 | 0 | 19.90 | thioredoxin H-type |
| Dgc4809, Dgc5686, Dgc6908 | 7 | 0 | 19.90 | COP9 signalosome complex subunit 5a-like |
| Dgc3606, Dgc3919, Dgc3810 | 7 | 0 | 19.90 | small nuclear ribonucleoprotein f |
| Dgc1192, Dgc2253 | 16 | 3 | 18.81 | proteasome subunit β type-1-like |
| Dgc80 | 18 | 4 | 17.38 | proline-rich protein (CAA42942) |
| Dgc1323, Dgc1373, Dgc4736, Dgc4825 | 13 | 2 | 15.19 | aquaporin PIP-2 |
| Dgc262, Dgc817 | 13 | 2 | 15.19 | NADP-dependent glyceraldehyde-3-phosphate dehydrogenase |
| Dgc833 | 10 | 1 | 13.71 | Adenine phosphoribosyltransferase |
| Dgc397 | 15 | 3 | 13.22 | glutamate synthase |
| Dgc1313, Dgc2921 | 3 | 14 | 12.73 | high mobility group protein B, DNA-binding protein MNB1B |
| Dgc213 | 17 | 4 | 12.50 | potassium transporter |
| Dgc2335, Dgc6131 | 6 | 0 | 11.96 | Interferon-induced GTP-binding protein Mx, dynamin family protein |
| Dgc2539, Dgc3428 | 6 | 0 | 11.96 | zinc finger A20 and AN1 domain-containing stress-associated protein 4 isoform |
| Dgc2352, Dgc5064 | 6 | 0 | 11.96 | SEC1 family transport protein SLY1 |
| Dgc3046, Dgc4062 | 6 | 0 | 11.96 | spermidine synthase |
| Dgc2703, Dgc5200 | 6 | 0 | 11.96 | acetolactate synthase, plastidic |
| Dgc2448, Dgc5197 | 6 | 0 | 11.96 | receptor protein kinase, serine/threonine-protein kinase At4g35230 |
| Dgc4904, Dgc5578 | 6 | 0 | 11.96 | white-brown-complex ABC transporter family |
| Dgc2715, Dgc3433 | 6 | 0 | 11.96 | heat shock transcription factor protein HSF8 |
| Dgc3244 | 6 | 0 | 11.96 | GTP binding protein, protein LURP-one-related 12 |
| Dgc2420 | 6 | 0 | 11.96 | heat shock protein 70 |
| Dgc1168 | 6 | 0 | 11.96 | putative ethylene-responsive element-binding protein |
| Dgc1331 | 6 | 0 | 11.96 | basic helix-loop-helix (bHLH) family transcription factor |
| Dgc2787 | 6 | 0 | 11.96 | cytochrome P450 705A20 |
| Dgc997 | 6 | 0 | 11.96 | vacuolar Ca2+ antiporter/cation exchanger |
| Dgc2675 | 6 | 0 | 11.96 | 5'-methylthioadenosine/S-adenosylhomocysteine nucleosidase |
| Dgc1173 | 6 | 0 | 11.96 | ABC transporter family protein |
| Dgc1475 | 6 | 0 | 11.96 | type 2 histone deacetylase |
| Dgc3097 | 6 | 0 | 11.96 | GTP-binding protein TypA/BipA homolog |
| Dgc1221 | 6 | 0 | 11.96 | cytochrome P450 |
| Dgc1166 | 6 | 0 | 11.96 | ribonucleotide reductase large subunit |
| Dgc2302 | 6 | 0 | 11.96 | peptidase M3 family protein / thimet oligopeptidase family protein |
| Dgc1141 | 6 | 0 | 11.96 | inactive rhomboid protein 1 |
| Dgc1348 | 6 | 0 | 11.96 | gag-pol polyprotein (affects translation) |
| Dgc1153 | 6 | 0 | 11.96 | speckle-type POZ protein (signal transduction) |
| Dgc2894 | 6 | 0 | 11.96 | cytochrome P450 705A5 |
| Dgc2184 | 6 | 0 | 11.96 | defensin-like protein, without the special C-terminal domain of DgDEF1/DgDEF2 |
| Dgc1182 | 6 | 0 | 11.96 | β-amyrin synthase |
| Dgc2596 | 6 | 0 | 11.96 | calcium-activated outward-rectifying potassium channel |
| Dgc3668 | 6 | 0 | 11.96 | pathogen-related protein-like CAN63863 |
| Dgc1519 | 6 | 0 | 11.96 | protein EXORDIUM like 3 |
| Dgc3121 | 6 | 0 | 11.96 | TATA-box-binding protein |
| Dgc2413 | 6 | 0 | 11.96 | BTB/POZ domain-containing protein At3g19850 |
| Dgc6358, Dgc6709, Dgc6516 | 6 | 0 | 11.96 | protein kinase family protein with ARM repeat domain |
| Dgc98, Dgs236, Dgc5202 | 42 | 19 | 11.05 | 60S ribosomal protein L5 |
| Dgc123, Dgc241 | 24 | 8 | 10.48 | 60S ribosomal protein L23A |
| Dgc919, Dgc2427 | 12 | 2 | 10.43 | S-adenosylmethionine synthase |
| Dgc501 | 12 | 2 | 10.43 | ATP synthase gamma chain, mitochondrial precursor |
| Dgc448 | 12 | 2 | 10.43 | gibberellin-regulated family protein (GAST1-like protein, GASA5-like protein) |
| Dgc831, Dgc1196 | 12 | 2 | 10.43 | COV1-like protein |

**Table S1B.** *Datisca glomerata* genes upregulated in roots compared to nodules. Genes mentioned in the manuscript and not published before are given in bold print.

| **454 contig/supercontig** | **Reads in nodules** | **Reads in roots** | **R value** | **Description of encoded protein** |
| --- | --- | --- | --- | --- |
| **Dgc34, Dgc498, Dgc218, Dgc550, Dgc1157, Dgc1440, Dgc1427, Dgc2454, Dgc1004, Dgc4880, Dgc6555, Dgc1198, Dgc961, Dgc439, Dgc961, Dgc439, Dgc653, Dgc605, Dgc515, Dgc2234, Dgc6198, Dgc1426, Dgs6105, Dgc5067, Dgs6106, Dgc890, Dgc1924, Dgc557** | 54 | 765 | 1.08E+119 | **metallothionein** |
| Dgc1 | 206 | 424 | 3.07E+012 | dgGHRP1 (dg200), homologue of ag164/agNT84 [1] |
| Dgc105 | 1 | 54 | 6.73E+10 | glycine-rich protein, Alba DNA/RNA binding protein |
| Dgc107 | 0 | 44 | 1.04E+10 | mitochondrial gene for large subunit ribosomal RNA |
| Dgc39, Dgc737, Dgc2470 | 0 | 33 | 2.82E+07 | cationic peroxidase |
| Dgc46, Dgc2098 | 0 | 28 | 1.92E+06 | gibberellin 20 oxidase |
| Dgc56, Dgc6846, Dgc382, Dgc477 | 6 | 48 | 6.72E+05 | patellin 1 (sec14-like protein) |
| Dgc497, Dgc5654, Dgc1208, Dgc4966 | 2 | 35 | 4.83E+05 | cytochrome P450, β-amyrin 24-hydroxylase |
| Dgc170, Dgs332 | 2 | 29 | 2.72E+04 | putative phi-1-like phosphate-induced protein |
| Dgc62 | 1 | 23 | 8.91E+03 | laccase |
| **Dgc87, Dgc1256 Dgc672, Dgc5947** | 11 | 47 | 4.69E+03 | **cysteine protease (papain type)** |
| Dgc407, Dgc803, Dgc2189 | 5 | 33 | 3.46E+03 | zinc finger A20 and AN1 domain-containing stress-associated protein 3-like, C2H2L domain class transcription factor |
| Dgc1257 | 0 | 16 | 2.96E+03 | pentatricopeptide repeat-containing protein |
| Dgc789 | 28 | 3 | 2.04E+03 | methylthioribose-1-phosphate isomerase |
| Dgc312, Dgc714, Dgs1291 | 1 | 20 | 2.03E+03 | hypersensitive-induced response protein 1 ABS01349 |
| Dgc335 | 0 | 15 | 1.78E+03 | FQR1 (flavodoxin-like quinone reductase 1) |
| Dgc384, Dgc835 | 0 | 14 | 1.04E+03 | aquaporin TIP subfamily |
| Dgc184 | 1 | 18 | 7.63E+02 | alcohol dehydrogenase |
| Dgc119 | 3 | 24 | 715.11 | minor allergen Alt a 7-like |
| Dgc101 | 2 | 21 | 662.63 | lupeol synthase, cycloartenol synthase, isomultifloreol synthase, β-amyrin synthase |
| Dgc1692 | 0 | 13 | 607.68 | isoflavone reductase |
| Dgc462, Dgc1855, Dgc1295 | 12 | 42 | 484.71 | glutathione S-transferase |
| Dgc1293 | 1 | 17 | 469.70 | AP2 domain-containing transcription factor |
| Dgc409 | 0 | 12 | 355.08 | late embryogenesis abundant protein-like, copper ion binding protein, putative structural constituent of cell wall, root cap antihaemostatic protein, root cap periphery gene 2 precursor |
| Dgc1978, Dgc4614 | 0 | 12 | 355.08 | peroxidase |
| Dgc707, Dgc2248, Dgc4307, Dgc6265 | 1 | 16 | 290.19 | charged multivesicular body protein 1 |
| Dgc191 | 1 | 16 | 290.19 | ubiquitin-conjugating enzyme E2 24-like |
| **Dgc2262, Dgc3426, Dgc3875** | 0 | 11 | 207.48 | **aspartic proteinase nepenthesin-2 precursor** |
| Dgc1579, Dgc2909 | 0 | 11 | 207.48 | SAUR family protein |
| Dgc2834, Dgc6662 | 0 | 11 | 207.48 | CASP-like protein |
| Dgc822, Dgc5275 | 1 | 15 | 179.88 | ETHYLENE-INSENSITIVE3 protein |
| Dgc330, Dgc1397 | 1 | 15 | 179.88 | ankyrin repeat-containing protein At3g12360-like |
| Dgc1720, Dgc647, Dgc6434, Dgc4427, Dgs5130 | 1 | 15 | 179.88 | Avr9/Cf-9 rapidly elicited protein, late embryogenesis abundant hydroxyproline-rich glycoprotein |
| Dgc240, Dgs1142 | 3 | 20 | 133.00 | LRR receptor-like serine/threonine-protein kinase At1g06840-like |
| Dgc403, Dgc702 | 3 | 20 | 133.00 | cinnamyl alcohol dehydrogenase |
| Dgc3419, Dgc5215, Dgc5830 | 0 | 10 | 121.23 | anthranilate N-benzoyltransferase, salutaridinol 7-O-acetyltransferase |
| Dgc879, Dgc2630 | 0 | 10 | 121.23 | peroxisomal membrane 22 kDa (Mpv17/PMP22) family protein |
| Dgc2518, Dgc5535 | 0 | 10 | 121.23 | flavone synthase II, cytochrome P450, family 712, subfamily A, polypeptide 1 |
| Dgc620, Dgc1652 | 0 | 10 | 121.23 | nuclear transport factor 2 isoform |
| Dgc727 | 0 | 10 | 121.23 | RNA recognition motif containing protein |
| Dgc805 | 0 | 10 | 121.23 | WRKY transcription factor |
| Dgc1280 | 0 | 10 | 121.23 | repressor of RNA polymerase III transcription MAF1 |
| Dgc495 | 0 | 10 | 121.23 | histone-lysine N-methyltransferase SUVR5 |
| Dgc559 | 0 | 10 | 121.23 | gibberellin 20 oxidase |
| Dgc1429 | 0 | 10 | 121.23 | anaphase-promoting complex subunit cdc20 |
| Dgc670 | 0 | 10 | 121.23 | xyloglucan endotransglycosylase/hydrolase precursor XTH-3 |
| Dgc1103, Dgc4046, Dgc5842 | 2 | 17 | 111.99 | Kunitz-type trypsin inhibitor,α-amylase/subtilisin inhibitor |
| Dgc618 | 1 | 14 | 111.92 | 1,4-glucan-protein synthase [UDP-forming] |
| **Dgc28** | 30 | 64 | 84.85 | **cysteine protease** |
| Dgc82 | 4 | 21 | 78.41 | aquaporin PIP1-1 |
| **Dgc132, Dgc5792, Dgc4857, Dgc6380, Dgc3546** | 8 | 29 | 77.79 | **papain-like cysteine proteinase** |
| Dgc698 | 0 | 9 | 70.84 | phosphate transporter |
| Dgc566 | 0 | 9 | 70.84 | PLATZ transcription factor family protein |
| Dgc685 | 0 | 9 | 70.84 | dopamine β-monooxygenase, auxin-responsive protein |
| Dgc1334 | 0 | 9 | 70.84 | BSD domain-containing protein |
| Dgc1120 | 0 | 9 | 70.84 | WRKY domain class transcription factor |
| Dgc1816, Dgc2534 | 0 | 9 | 70.84 | secretory peroxidase |
| Dgc164 | 3 | 18 | 59.15 | calmodulin |
| Dgc3862, Dgc680, Dgc4766, Dgs5098 | 2 | 15 | 47.44 | ammonium transporter AMT1;2 |
| Dgc1597, Dgc3038, Dgc6560, Dgc3828, Dgc4998 | 1 | 12 | 43.90 | 3-hydroxy-3-methylglutaryl coenzyme A reductase |
| Dgc441 | 1 | 12 | 43.90 | 26S proteasome non-ATPase regulatory subunit 12 |
| Dgc490 | 1 | 12 | 43.90 | hydroxymethylglutaryl-CoA synthase |
| Dgc417, Dgc1034 | 1 | 12 | 43.90 | dual specificity protein kinase PYK1 |
| Dgc832 , Dgc1271, Dgc2830, Dgc3064 | 27 | 7 | 42.89 | 60S ribosomal protein L19 |
| Dgc701 | 0 | 8 | 41.39 | mitochondrial dicarboxylate carrier protein |
| Dgc818 | 0 | 8 | 41.39 | methylenetetrahydrofolate reductase (NADPH) |
| Dgc736 | 0 | 8 | 41.39 | trans-cinnamate 4-hydroxylase |
| Dgc745 | 0 | 8 | 41.39 | lactoylglutathione lyase |
| Dgc743 | 0 | 8 | 41.39 | phosphate transporter |
| Dgc629 | 0 | 8 | 41.39 | brassinosteroid-regulated protein BRU1 precursor |
| Dgc884 | 0 | 8 | 41.39 | CASP-like protein |
| Dgc1083 | 0 | 8 | 41.39 | indole-3-acetic acid amido synthetase |
| Dgc244 | 3 | 17 | 39.83 | dehydroascorbate reductase |
| Dgc79 | 5 | 21 | 36.33 | mitochondrial phosphate transporter |
| Dgc744 | 1 | 11 | 27.72 | RING finger and CHY zinc finger domain-containing protein |
| Dgc1609, Dgc1845, Dgc3001 | 1 | 11 | 27.72 | NAC transcription factor |
| Dgc561 | 3 | 16 | 27.00 | saposin B domain-containing protein |
| Dgc3360, Dgc4829 | 0 | 7 | 24.19 | importin subunit α-1 |
| Dgc1863, Dgc3228 | 0 | 7 | 24.19 | protein binding / zinc ion binding, ubiquitin-protein ligase-like |
| Dgc2278 | 0 | 7 | 24.19 | cationic peroxidase |
| Dgc996 | 0 | 7 | 24.19 | SelT-like protein precursor |
| Dgc986 | 0 | 7 | 24.19 | putative pre-mRNA splicing factor |
| Dgc1641 | 0 | 7 | 24.19 | putative transcription elongation factor SPT5 homolog 1-like isoform |
| Dgc1131 | 0 | 7 | 24.19 | protease inhibitor/seed storage/lipid transfer protein (LTP) family protein, extensin-like protein |
| Dgc941 | 0 | 7 | 24.19 | calcium-binding protein CML36-like |
| Dgc965 | 0 | 7 | 24.19 | actin |
| Dgc3205 | 0 | 7 | 24.19 | macrophage erythroblast attacher-like |
| Dgc2127, Dgs2876 | 0 | 7 | 24.19 | receptor protein kinase At1g67000-like |
| Dgc389, Dgc466 | 9 | 27 | 24.04 | heavy-metal-associated domain-containing protein |
| Dgc277 | 2 | 13 | 20.63 | syntaxin-121 |
| Dgc187 | 2 | 13 | 20.63 | secretory peroxidase 3 |
| Dgc438 | 2 | 13 | 20.63 | blue copper protein, cupredoxin-like, ENOD16 like protein |
| Dgc1035 | 2 | 13 | 20.63 | dehydration-induced protein ERD15, polyadenylate-binding protein-interacting protein |
| Dgc147, Dgc3376, Dgc1216, Dgc1511 | 12 | 31 | 18.77 | asparaginyl endopeptidase; vacuolar processing enzyme-1b; cysteine protease |
| Dgc178, Dgc652 | 3 | 15 | 18.46 | 2-oxoglutarate-dependent dioxygenase |
| Dgc52 | 14 | 34 | 18.31 | flavonoid 4'-O-methyltransferase, caffeic acid O-methyltransferase |
| Dgc77, Dgc1138, Dgc1241, Dgc1953 | 4 | 17 | 17.95 | glutathione S-transferase |
| Dgc711, Dgc2032 | 1 | 10 | 17.62 | squalene synthase, farnesyl-diphosphate farnesyltransferase |
| Dgc601 | 1 | 10 | 17.62 | ATP-citrate lyase/succinyl-CoA ligase |
| Dgc556 | 1 | 10 | 17.62 | PIP1 aquaporin |
| Dgc1534, Dgc2947, Dgc2577 | 1 | 10 | 17.62 | 4-hydroxyphenylpyruvate dioxygenase |
| Dgc1869, Dgc4315 | 0 | 6 | 14.13 | gibberellin receptor GID1 |
| Dgc1343 | 0 | 6 | 14.13 | β-ketoacyl-ACP synthase III |
| Dgc1415 | 0 | 6 | 14.13 | 3-hydroxy-3-methylglutaryl-coenzyme A reductase |
| Dgc1252 | 0 | 6 | 14.13 | glutathione S-transferase GST 23 |
| Dgc1205 | 0 | 6 | 14.13 | short-chain dehydrogenase/reductase |
| Dgc1099 | 0 | 6 | 14.13 | basic-leucine zipper transcription factor bZIP69 |
| Dgc932 | 0 | 6 | 14.13 | chromatin remodeling complex subunit, SWI/SNF complex subunit SWI3D, ras-related protein RAB7C |
| Dgc1069 | 0 | 6 | 14.13 | glutaredoxin family protein |
| Dgc1268 | 0 | 6 | 14.13 | Ser/Thr protein kinase YAK1 |
| Dgc1139 | 0 | 6 | 14.13 | ras-related small GTP-binding protein |
| Dgc2280 | 0 | 6 | 14.13 | ubiquitin-conjugating enzyme E2 |
| Dgc1583 | 0 | 6 | 14.13 | cyclic nucleotide-gated ion channel |
| Dgc953 | 0 | 6 | 14.13 | C2H2L domain class transcription factor |
| Dgc1721 | 0 | 6 | 14.13 | MLO protein (plasmamembrane protein, calmodulin binding, defense response) |
| Dgc2285 | 0 | 6 | 14.13 | carbon catabolite repressor protein 4 |
| Dgc1191 | 0 | 6 | 14.13 | rhamnogalacturonate lyase family protein |
| Dgc323 | 2 | 12 | 13.77 | polygalacturonase precuror |
| Dgc54 | 9 | 25 | 13.53 | SNAP protein |
| Dgc1313, Dgc2921 | 3 | 14 | 12.73 | high mobility group protein B, DNA-binding protein MNB1B |
| Dgc2122, Dgc4691 | 1 | 9 | 11.28 | E3 ubiquitin ligase PUB14, U-Box domain containing protein |
| Dgc1611, Dgc2815, Dgc3693 | 1 | 9 | 11.28 | actin depolymerizing factor |
| Dgc773 | 1 | 9 | 11.28 | rapid alkalinization factor precursor |
| Dgc945 | 1 | 9 | 11.28 | isocitrate dehydrogenase [NAD] regulatory subunit 1, mitochondrial |
| **Dgc258, Dgc2439, Dgc1256, Dgc2487, Dgc5947, Dgc2254, Dgc1422, Dgc826, Dgc3820** | 15 | 33 | 10.03 | **cysteine protease** |

**References**

1. Pawlowski K, Swensen S, Guan C, Hadri A-E, Berry AM, Bisseling T (2003) Distinct patterns of symbiosis-related gene expression in actinorhizal nodules from different plant families. Mol Plant-Microbe Interact 16: 796-807.
2. Okubara PA, Fujishige NA, Hirsch AM, Berry AM (2000) *Dg93*, a nodule-abundant mRNA of *Datisca glomerata* with homology to a soybean early nodulin gene. Plant Physiol 22: 1073-1079.
3. Ribeiro A, Akkermans ADL, van Kammen A, Bisseling T, Pawlowski K (1995) A nodule-specific gene encoding a subtilisin-like protease is expressed in early stages of actinorhizal nodule development. Plant Cell 7: 785-794.
4. Berry AM, Murphy TM, Okubara PA, Jacobsen KR, Swensen SM, Pawlowski K (2004) Novel expression pattern of cytosolic glutamine synthetase in nitrogen-fixing root nodules of the actinorhizal host, *Datisca* *glomerata*. Plant Physiol 135: 1849-1862.
5. Jeong J, Suh SJ, Guan C, Tsay Y-F, Moran N, Oh CJ, An CS, Demchenko K, Pawlowski K, Lee Y (2004) A nodule-specific dicarboxylate transporter from *Alnus glutinosa*. Plant Physiol 134: 969-978.
6. Pawlowski K, Jacobsen KR, Alloisio N, Denison RF, Klein M, Winzer T, Sirrenberg A, Guan C, Berry AM (2007) Truncated hemoglobins in actinorhizal nodules of *Datisca glomerata.* Plant Biol 9: 776-785.
