# Supplementary material for: Comparison of the Nodule vs. Root Transcriptome of the Actinorhizal Plant Datisca glomerata: Actinorhizal Nodules Contain a Specific Class of Defensins
Source: PLoS One. 2013 Aug 29;8(8):e72442. doi: 10.1371/journal.pone.0072442 (PMC3756986; doi:10.1371/journal.pone.0072442)
Supplement: Table S2 — Homology between legume proteins (GenBank accession numbers given) involved in nodule induction, and the corresponding proteins encoded by Datisca glomerata nodule contigs. The Lotus japonicus (Lj) and/or Medicago truncatula (Mt) protein sequences were used for a tBlastN search on the different assemblies of the D. glomerata nodule transcriptome. The homology values (E values) for the contigs given in Table 3 are listed. Homologies for D. glomerata SYMRK/DMI2 are not given as this gene was already functionally characterized [33]. (DOC) [file pone.0072442.s005.doc]

**Table S2.** Homology between legume proteins (GenBank accession numbers given) involved in nodule induction and the corresponding proteins encoded by *Datisca glomerata* nodule contigs. The *Lotus japonicus* (Lj) and/or *Medicago truncatula* (Mt) protein sequences were used for a tBlastN search on the different assemblies of the *D. glomerata* nodule transcriptome. The homology values (E-values) for the contigs given in Table 3 are listed. Homologies for *D. glomerata* SymRK/DMI2 are not given as this gene was already functionally characterized [1].

| **Protein name** | ***L. japonicus*** | ***M. truncatula*** | ***Dg* contig** | **E (Lj)** | **E (Mt)** |
| --- | --- | --- | --- | --- | --- |
| NFR1/LYK3 | CAE02590 | CAM06621 | comp2293_c1_seq4 | 0 | 0 |
| NFR5/NFP | CAE02597 | ABF50224 | comp7396_c0_seq1 | 4.00E-146 | 3.00E-154 |
| CCaMK/DMI3 | CAJ76700 | Q6RET7 | comp5284_c0_seq1 | 0 | 0 |
| CASTOR/DMI1 | BAD89021 | AAS49490 | comp9681_c0_seq9 | 0 | 0 |
|  |  |  | comp8316_c0_seq1 | 0 | 0 |
| POLLUX | BAD89022 | - | comp9681_c0_seq10 | 0 | - |
|  |  |  | comp8316_c0_seq1 | 0 | - |
| RIT/NAP1 | CAQ17048 | AES90091 | comp4244_c0_seq2 | 0 | 0 |
| PIR1 | - | XP_003626502 | comp3407_c0_seq1 | - | 0 |
| SYMREM1 | - | AEX20500 | comp279_c0_seq1 | - | 3.00E-41 |
|  |  |  | comp115_c0_seq1 | - | 6.00E-40 |
| NUP133 | CAI64811 | - | comp6865_c0_seq1 | 0 | - |
| CYCLOPS/IPD3 | ABU63668 | ABN45743 | comp2070_c0_seq4 | 6.00E-115 | 5.00E-125 |
| NIN | CAB61243 | ACN58567 | comp564_c0_seq1 | 0 | 0 |
| ERN1 | - | ABW06102 | comp6569_c0_seq1 | - | 3.00E-55 |
| NSP1 | ABK35066 | CAJ00005 | comp755_c1_seq1 | 0 | 0 |
| NSP2 | ABG49438 | XP_003601076 | comp1841_c1_seq1 | 0 | 4.00E-154 |
| HAP2-1 |  | ABP68866 | comp860_c0_seq1 | - | 1.00E-80 |
| LIN/CERBERUS | C6L7U1 | ACL14420 | comp2398_c0_seq12 | 0 | 0 |
| HK1/CRE1 | ABI48271 | XP_003617960 | comp11620_c0_seq2 | 0 | 0 |
|  |  |  | comp6496_c0_seq2 | 0 | 0 |
|  |  |  | comp1838_c0_seq4 | 0 | 0 |
|  |  |  | comp13545_c0_seq2 | 4.00E-150 | 0 |
| HMGR1 | - | ABY20972 | comp406_c0_seq4 | - | 0 |
|  |  |  | comp3746_c0_seq1 | - | 0 |
| VPY | - | ADC33495 | comp804_c0_seq1 | - | 0 |
| PUB1 | - | DAA33939 | comp11879_c0_seq4 | - | 0 |

**References**

1. Markmann K, Giczey G, Parniske M (2008) Functional adaptation of a plant receptor-kinase paved the way for the evolution of intracellular root symbioses with bacteria. PLoS Biol 6: e68.
