# Supplementary material for: Comparison of the Nodule vs. Root Transcriptome of the Actinorhizal Plant Datisca glomerata: Actinorhizal Nodules Contain a Specific Class of Defensins
Source: PLoS One. 2013 Aug 29;8(8):e72442. doi: 10.1371/journal.pone.0072442 (PMC3756986; doi:10.1371/journal.pone.0072442)
Supplement: Table S3 — Preparation of cDNA libraries (1, root cDNA library; 2, nodule cDNA library). PCR cycles used for cDNA amplification and barcode sequences attached to 5′-ends of cDNAs (DOCX) [file pone.0072442.s006.docx]

**Table S3.** Preparation of cDNA libraries (1, root cDNA library; 2, nodule cDNA library). PCR cycles used for cDNA amplification and barcode sequences attached to 5'-ends of cDNAs.

| cDNA library | 1 | 2 | |
| --- | --- | --- | --- |
| RNA sample | roots | 1/3 young nodules | 2/3 mature nodules |
| 5’-barcode | ACTA | CAGC | |
| PCR cycles | 13 | 13 | |
