# Supplementary material for: Comparison of the Nodule vs. Root Transcriptome of the Actinorhizal Plant Datisca glomerata: Actinorhizal Nodules Contain a Specific Class of Defensins
Source: PLoS One. 2013 Aug 29;8(8):e72442. doi: 10.1371/journal.pone.0072442 (PMC3756986; doi:10.1371/journal.pone.0072442)
Supplement: Table S4 — Primers used for amplification of cDNAs from Datisca glomerata. (DOCX) [file pone.0072442.s007.docx]

1. **Table S4.** Primers used for amplification of cDNAs from *Datisca glomerata.*

| PCR | Primers |
| --- | --- |
| *DgDCAT1* (*Dgc108*) |  |
| First 5’-RACE | **CCACTCACCACCCACCGATCGATCCC**  **GCTCGTGCCACTACCACCTTCACC** |
| Second 5’-RACE | **CGTTGTCGTTGCTACTTGACTGCCA**  **CAACCGCTGCACGGTCCAAGAATC** |
| 3’-RACE | **GGTGAGTGGTGGGTGGCTAGCTAGGG**  **CGAGCACGGTGATGGTGGAAGTGG** |
| Full-length cDNA amplification | **ACGCGGGACAAAGCAGCA**  **GGAATGATGAGAGCTCTGATTCACCAAAAT** |
| Sequencing | **ACTACCCATTCCGGAGAATGACG**  **TGGAGAAAGTTTGGAGTTGAGCC**  **GGAGGTGAAACAAAGTGATGGGTG** |
| *DgDEF1* (*Dgc156*) |  |
| 5’-RACE | **CTCAGGGAAATCCCAGATAGAAG**  **GACATTATGCATTGC** |
| Full-length cDNA amplification | **GACATCAACTGCTCAAGTCATCG**  **TGCATTGCATGACACAAACAT** |
| *DgCRP1* (*Dgc232*) |  |
| First 5’-RACE | **AGAGTACGTGAGGTACCTACTACATTGG**  **GTCGAGTCACTCTACTTACC** |
| Second 5’-RACE | **GGAGCTTCAGAAGGAGGATAAGATGGA**  **AATGATCCCTTCTTGCCACTTCCTTCC** |
| 3’-RACE | **TGGAGTCTTGCCCTGGTTTTTGTGC**  **ATCCCAATGTAGTAGGTACCTCACGTAC** |
| Full-length cDNA amplification | **CATCAAACAATTAGAAACAAATTTCATC**  **TATTTGCAATTGACATTGCAAAACTATC** |
| *Dg768* (*Dgc768*) |  |
| 5’-RACE | **ACGACAAGGGTATAACAAGAACATAGC**  **AGCTCAAACAAGTATAGATATCCCATGT** |
| 3’-RACE | **CCATGCTGGACAAAGTCTATCCCACT**  **GCCATGTGCTGGCAATATCTTTTCTTG** |
| Full-length cDNA amplification | **CGGGGAACCCACCTAAAT**  **TTTACAAAGTAAAACTTTTTCATTACCGT** |
| *DgMnSOD1* (*Dgc73*) |  |
| 5’-RACE | **GGTTTGCACTGCAGTCCTTGCCA**  **CTGGCCTGACGTTTTTGTACTGT** |
| 3’-RACE | **GGGCTCTGGATGGGTGTGGCTTGC**  **CGGCTAATCAGGATCCCCTTGTGACCA** |
| *DgDEF2 (Dgc 845)* |  |
| Full-length cDNA amplification | **ATGGCTAACACTCCTAAAGACATTCCTC**  **ATTGCAATCAAACTTGAATTTCCTCCA** |

| PCR | **Primers** |
| --- | --- |
| *DgPUB1 (Comp11879)* |  |
| Full-length cDNA amplification | **TGACGCAGTGAGAGTGCAACATTGG**  **ATACTTCTGCAATTCCTCTACCGTCTTC** |
| Sequencing | **TCGTTGGTTCCAATCCCGGAC**  **TTCTTCTGCTGTTGCTTCTCTCC**  **TCTGACTTGAACAAAAGGCATGT**  **CTGATGATGCATTGCAAGTGCT** |
| *DgNIN1 (Dgc1007)* |  |
| Full-length cDNA amplification | **GCCAACAATTCTCCCACAAGAGATCG**  **GTAATGGTTTCAGGACGGGCTGC** |
| Sequencing | **GGTCAGCAGGTTGATGTTAGG**  **CTTTCCTTCCCCCTGACGA**  **GGACAAGTATTTTCAGAATTTGGACAG**  **TCGAAAACAGGCAATGCCA** |
| *DgVPY (Dgc3012)* |  |
| Full-length cDNA amplification | **CCATTTCTGCCATCTCTCACTCCTCT**  **TATGAAAGTTTGGTGCAAATTGTCACTCTG** |
| Sequencing | **GAGGTTCTGAGTCTTCTCCTGC**  **GTGCTCATCCCCTACTCTAGCC** |
| *DgREM1 (Dgc390)* |  |
| Full-length cDNA amplification | **TGAAGTAACTTTGAGAATTGCAGA**  **GGAGCAGAGTTTTATTTAGGCTG** |
| *DgREM2 (Dgc995)* |  |
| Full-length cDNA amplification | **GTGGCTAAGCTGAAAGAGTACG**  **CATGTTTCTCACACATCCATCTG** |
