# Supplementary material for: Comparison of the Nodule vs. Root Transcriptome of the Actinorhizal Plant Datisca glomerata: Actinorhizal Nodules Contain a Specific Class of Defensins
Source: PLoS One. 2013 Aug 29;8(8):e72442. doi: 10.1371/journal.pone.0072442 (PMC3756986; doi:10.1371/journal.pone.0072442)
Supplement: Table S5 — Primers used in quantitative real-time PCR. (DOCX) [file pone.0072442.s008.docx]

**Table S5.** Primers used in quantitative real-time PCR.

| Gene | Forward primer | Reverse primer |
| --- | --- | --- |
| ***Dgc11*** | CCGATTCTCGGACCAGCAG | TGTGTTCGTTCTATTCTGATGGATTAC |
| ***Dgc26*** | CTCGTGGCAATAAACGCAAG | AACACACACATCTTCAAACTTTCATC |
| ***Dgc39*** | GAAATAAGGAAGAATTGCAGGGTTG | AACATCAAATCCAAAATCACACATAAA |
| ***Dgc54*** | TCTATGTCGATGTGGAAGAGATTGATG | GGGATGGGGAAAAGGCAAAG |
| ***DgMnSOD1*** | GCGGAGGTCATATAAACCACTC | AGAGCAAGCCACACCCATCC |
| ***Dgc80*** | TCTCCATGGCGGATTGAAGG | TCAACGTGGGCAGGAAGGTT |
| ***Dgc87*** | ACCGTTGCTTCTGTTCATACTACCTC | GCTAGCCATAAAAAGTATGTAGCATTG |
| ***DgDCAT1*** | GGCAGTAGCCGGTTTCGTTG | AAACTCCATCAGCCCCACCA |
| ***Dgc148*** | ATGCCACCCGATGCACAA | TGCCATCTTAGCGTTGATCCTG |
| ***DgDEF1*** | TCCATTTTCATTACCTTGCTTCTCC | TTCTCCCATCTCCGGCATCT |
| ***Dgc205*** | CATGAACATGCAGATCTTCGTGA | AGCACCAGGTGAAGGGTCG |
| ***DgCRP1*** | TGGAAGGAAGTGGCAAGAAGG | TGGCAGAAGAACCATACCAGCA |
| ***Dgc323*** | CGCGTGCAGTGACAGTTTCC | TCTGCCAACAGAAAGGATCAAGC |
| ***Dgc556*** | AGAGCCATTCCTTTCAAGTCCAG | GCTCAACACCAAAATACAATCAAAGAA |
| ***Dgc670*** | CATGCGTAAGATTCCGTCAAAAGTTA | ATAATCACACATTCCGGTGGTGGA |
| ***Dgc768*** | CAAAGATCAGCCAAACAAATCTGG | AGAAAAGATATTGCCAGCACATGG |
| ***Dgc970*** | CAACATGCGGTGGCTTTG | AGAGAGAAAGAGAGAGTTGTGATCATATT |
| ***Dgc1083*** | GGTGCATCTATCAACCAATACAAGGT | GCGTTCTGGGGTCCAATGT |
| ***Dgc1131*** | GCTGCTGTTTGCCTTTGCACT | TGAAACTCAGGTGGAGTCTTTTTACCA |
| ***Dgc1139*** | CGACAGTACAGATTCGCGGACA | TGCACACAGGTAAACATCACTAAATCA |
| ***Dgc1305*** | CATGGAAGAAGTAAACTGGGCAAA | CCTTTGTTCTCCAGATGTTTGTGTG |
| ***DgDEF2*** | TGCAGCTCATGGATTATGTCAAAGA | TGCCATAGCCACCTTCTCCAA |
| ***DgNIN1*** | CAAAGTCCCCCACCTCCTCA | ATCCCGATCCGTCCTGTCAC |
| ***DgREM1*** | TGGCAGAGGAAGAACCCAAGA | TCAGCAGGGGGAGGTGGA |
| ***DgREM2*** | TGCAGGTGGTTCCATTGACAG | TTTTCGCTTTCCTCCCATGC |
| ***DgPUB1*** | GTTGTGGTTGCTGGGGCAGT | CCTGAAGCCCGTCAGAGCA |
| ***DgCERBERUS*** | TTTGCTGGCTGTTCTTCAGTCG | CGGCTATGCGATGGATGTCA |
| ***DgVPY1*** | ACGGCTCTTCACCTCGCAGT | TCTAGCCGCGATATGCAACG |
